# Nature‐Inspired Design Strategies for Efficient Atmospheric Water Harvesting

**DOI:** 10.1002/adma.202519362

**Published:** 2025-11-10

**Authors:** Yunchan Lee, Shouhong Fan, Shu Yang

**Affiliations:** ^1^ Department of Materials Science and Engineering University of Pennsylvania 3231 Walnut Street Philadelphia PA 19104 United States

**Keywords:** atmospheric water harvesting, bioinspiration, fog harvesting, nanomaterials, sorption‐based atmospheric water harvesting, water scarcity

## Abstract

The accelerating global scarcity of freshwater, driven by rapid economic growth, environmental degradation, and climate change, has heightened the urgency for the development of advanced atmospheric water harvesting (AWH) strategies. Earth's atmosphere contains an enormous reservoir of ≈13 trillion tons of water, exceeding the volume of all accessible surface freshwater, yet it remains vastly underutilized. Effectively utilizing the atmospheric water requires efficient capture and release strategies that can function under diverse climatic conditions. Through millions of years, organisms in both arid and humid environments have evolved specialized structures or surfaces to capture water directly from the atmosphere. This review highlights recent advances in bioinspired AWH systems, emphasizing how structural motifs such as wettability gradients, directional transport, and hierarchical porosity are translated into engineered fog‐collection and vapor‐sorption systems, leading to enhanced water uptake, accelerated transport, and energy‐efficient release. By integrating insights from various complementary approaches, the design principles and fabrication strategies that bridge biological inspiration with practical, high‐efficiency AWH for scalable solutions to address the global water challenges are outlined.

## Graphical abstract

This review presents advances in bioinspired atmospheric water harvesting systems, emphasizing how structural motifs such as wettability gradients, directional transport, and hierarchical porosity have been translated into engineered fog‐collection and vapor‐sorption strategies for enhanced water uptake, accelerated transport, and energy‐efficient release. It outlines the design principles that bridge biological inspiration with practical, scalable solutions to address the global water challenges.

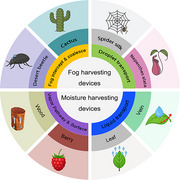

## 1 Introduction

Water is one of Earth's most abundant and essential resources, integral to sustaining human societies and natural ecosystems.^[^ ^1^ ^]^ Exacerbated by the rapid population growth, industrialization, and climate change, water scarcity has emerged as one of the most pressing global challenges.^[^ ^2^, ^3^, ^4^ ^]^ Various remedy strategies have been developed, including seawater desalination, rainfall harvesting, groundwater extraction, and atmospheric moisture condensation.^[^ ^5^, ^6^, ^7^, ^8^, ^9^, ^10^ ^]^ However, they often involve high capital and operational expenditures, significant energy consumption, or geographic constraints, making them less practical for remote areas such as deserts, plateaus, and mountainous regions. Large‐scale water diversion projects can also lead to environmental degradation, land loss, and heightened vulnerability to contamination.^[^ ^11^, ^12^, ^13^ ^]^ As a result, capturing water from more sustainable sources has been a key focus of research over the last decade. In this context, atmospheric water harvesting (AWH) has gained increasing attention as a decentralized and sustainable pathway to provide clean water.^[^ ^14^, ^15^ ^]^ The atmosphere contains an enormous reservoir of ≈13 trillion tons of water,^[^ ^14^ ^]^ greater than all accessible surface freshwater combined, yet this ubiquitous resource remains vastly underutilized.

Fog‐ and vapor‐based water harvesting represent the most promising strategies among the current AWH approaches. Fog harvesting captures tiny water droplets floating in the air and turns them into liquid water that can be collected, making it particularly suitable for coastal or highland regions with frequent fog events.^[^ ^16^, ^17^, ^18^ ^]^ By contrast, sorption‐based atmospheric water harvesting (SAWH) relies on hygroscopic materials to extract vapor even under low relative humidity (RH) conditions in arid regions such as deserts, followed by controlled release, thereby offering broader geographical applicability.^[^ ^15^, ^19^ ^]^ Despite their respective advantages, both strategies face critical limitations. Fog collectors depend highly on specific climatic conditions, exhibit low efficiency under low fog density, and often suffer durability issues. While SAWH systems are less restricted by geographic or climatic conditions, they remain constrained by slow water uptake kinetics, high regeneration energy demand, and durability issues such as salt leakage from hygroscopic materials. These challenges highlight that adequate progress in AWH requires material optimization and structural design principles that can bridge different mechanisms.

Nature provides a compelling blueprint. Over millions of years, organisms have evolved highly specialized structures to extract water directly from the atmosphere at a broad range of RH.^[^ ^20^, ^21^, ^22^, ^23^ ^]^ For example, an array of bumpy, waxy hemispheres can be found on the scales of Namib Desert beetles. When the beetle's body tilts, the fog droplets accumulate and drip into its mouth.^[^ ^24^, ^25^ ^]^ The cacti capture fog on conical spines, leveraging Laplace pressure and surface wettability gradients to transport droplets toward water‐absorbing trichomes.^[^ ^26^, ^27^ ^]^ The spider silk features periodic spindle‐knots that generate directional droplet movement through a combination of surface energy gradients and curvature‐induced Laplace pressure.^[^ ^28^, ^29^ ^]^ The Nepenthes alata pitcher plant utilizes a specialized structure with a micro‐ and nano‐grooved surface that becomes superhydrophilic when wetted, creating a slippery, continuous water film. This film drastically reduces surface friction, causing impinging water droplets to spread rapidly and be guided along radially oriented channels toward the pitcher's interior.^[^ ^30^, ^31^ ^]^ Tillandsia plants rely on specialized trichomes that directly capture atmospheric water and channel it into storage tissues.^[^ ^32^, ^33^ ^]^ Vascular wood utilizes hierarchical xylem channels to regulate capillary‐driven transport with directional efficiency.^[^ ^34^ ^]^ These examples showcase how organisms integrate micro‐ and nano‐scale structures with chemical heterogeneity to harvest atmospheric moisture, whether as fog droplets or water vapor, through optimized capture, absorption, transport, and storage mechanisms.

**Figure 1:**
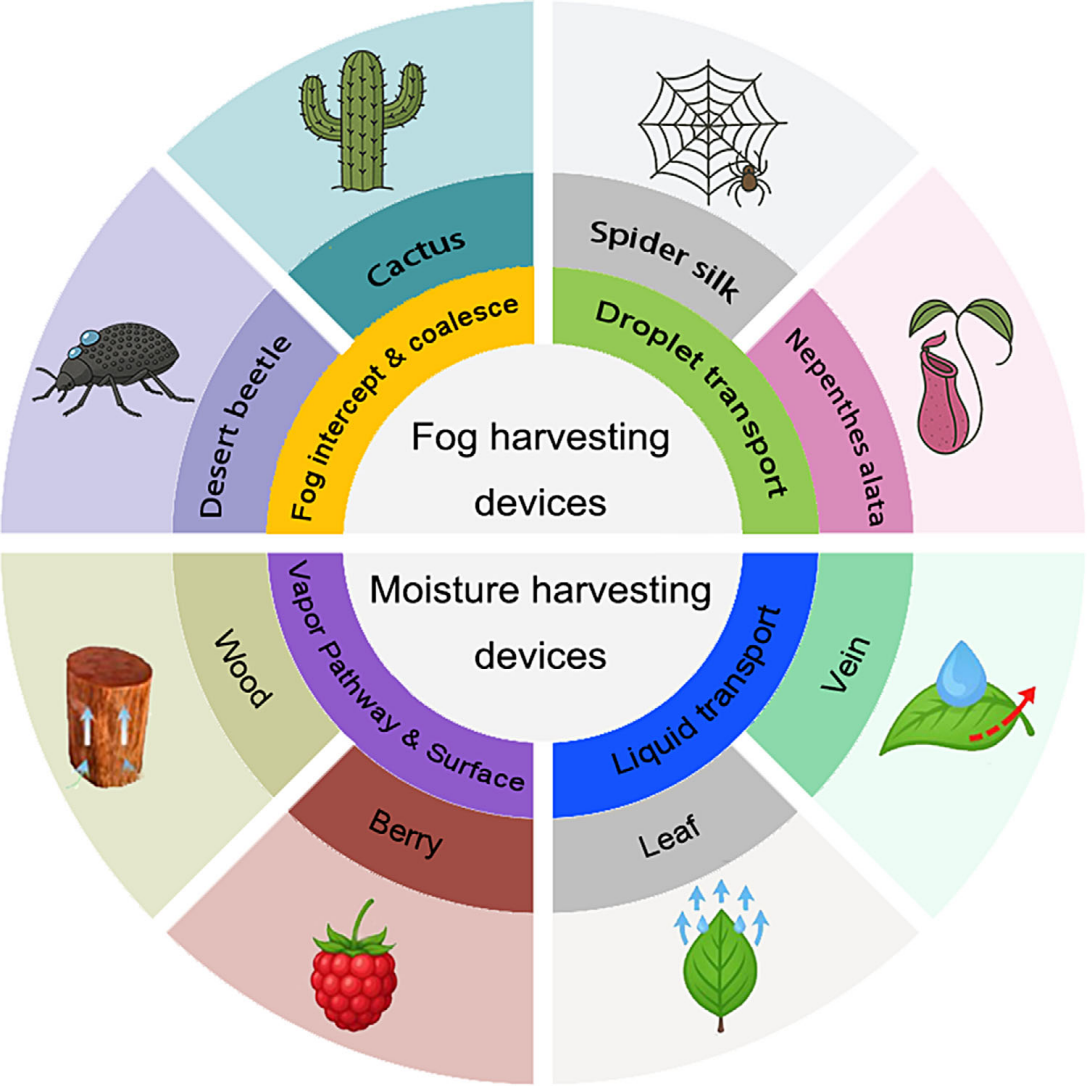
Schematic illustration of nature's examples and working mechanisms for fog and moisture harvesting.

This review discusses a nature‐inspired structural framework that bridges fog collection and SAWH strategies (**Figure** **1**). First, we examine how bioinspired structural and surface designs, such as wettability gradients, surface curvature, anisotropic textures, and hierarchical topographies, have been implemented in fog‐harvesting systems to facilitate droplet capture and transport. Next, we outline different classes of hygroscopic materials for SAWH and their working principles, highlighting how nature‐inspired architectures have been integrated to accelerate water uptake, reduce diffusion barriers, and enable efficient release of water from hygroscopic materials. Finally, we discuss remaining challenges and outline future directions for developing efficient, sustainable, nature‐inspired AWH technologies capable of addressing water scarcity in diverse environments.

## 2 Nature‐Inspired Structures for Efficient Fog Harvesting

### 2.1 Theoretical Basis

The process of fog harvesting on a surface involves a sequence of interrelated steps: microscopic airborne droplets are first intercepted and adhered to the collection surface, then the droplets coalesce into larger drops that migrate across the substrate, and finally detach guided by wettability gradient, Laplace pressure gradient, or liquid–gas interfacial tension.^[^ ^35^ ^]^ Here, the wettability gradient refers to surfaces that have spatial variations in surface energy, leading to a transition from hydrophobic (low surface energy) to hydrophilic (high surface energy). Laplace pressure arises when water droplets form on surfaces with varying curvatures from one side of the material to another side, such as a conical spine. This curvature‐induced pressure gradient spontaneously drives droplet motion from regions of high curvature (high Laplace pressure) to regions of low curvature (low Laplace pressure). Liquid–gas interfacial tension is the energy per unit area associated with the interface between a liquid and a gas, arising from the imbalance of molecular forces at the boundary.^[^ ^36^ ^]^ The efficiency of this process is governed by a combination of three fundamental parameters: aerodynamics, surface geometry, and surface wettability, which are discussed in detail in the following sections.

Once fog is captured on the surface of fog‐harvesting materials, it first nucleates on the surface, followed by coalescing into large droplets. From a theoretical standpoint, nucleation follows the principle of classical nucleation theory, where condensation requires overcoming an energy barrier to form a stable liquid nucleus from vapor.^[^ ^37^, ^38^ ^]^ For homogeneous nucleation, this barrier is high because droplets must form spontaneously in bulk vapor. In contrast, heterogeneous nucleation occurs on a solid surface and requires significantly less energy because the solid–liquid interface lowers the Gibbs free energy (Δ*G*) barrier by reducing the critical nucleus size.^[^ ^39^ ^]^ The Δ*G* is expressed as

(1) $$\Delta G=\pi \sigma_{IV}r{*}^{2}\left(2-3cos\theta +cos^{3}\theta \right)/3$$

where σ_*IV*_ is the liquid–vapor surface energy, r* is the critical radius, and θ is the static contact angle of water. The r* is given by Kelvin's classical equation,

(2) $$\ln\left(\frac{P}{P_{\infty }}\right)=\frac{2\sigma_{IV}}{n_{l}kTr*}$$

where *P* is the vapor pressure over a curved interface, between the droplet and the vapor, *P*_∞_ is the equilibrium vapor pressure above a flat surface of the condensed droplet at a temperature of *T*, *n_l_* is the molar volume corresponding to the number of molecules per unit volume of the liquid, and *k* is the Boltzmann constant. The degree of reduction depends on the θ of the condensing phase on the surface, expressed by a geometric factor

(3) $$f\left(\theta \right)=\frac{\left(2+cos\theta \right){\left(1-cos\theta \right)}^{2}}{3}$$

which modifies the critical free energy. Hydrophilic regions (low θ) strongly promote nucleation by minimizing the energy barrier, leading to a faster onset of droplet condensation through nucleation and growth.^[^ ^39^ ^]^ Specifically, the nucleation rate (*J*)via has an inverse exponential dependence on the Δ*G*, given by

(4) $$J=J_{0}\exp\left(-\frac{\Delta G}{kT}\right)=J_{0}\exp\left[\frac{\pi \sigma_{IV}r{*}^{2}\left(2-3cos\theta +cos^{3}\theta \right)}{3kT}\right]$$

where *J*_0_is a kinetic constant.

As water droplets condense from the fog through nucleation and growth, they must be transported efficiently toward a reservoir to prevent clogging, surface flooding, and re‐evaporation, which ultimately ensures the materials’ water harvesting efficiency from the fog. Natural systems employ passive directional water transport, driven by surface wettability gradient, Laplace pressure differences, and liquid–gas interfacial tension asymmetries.

**Figure 2:**
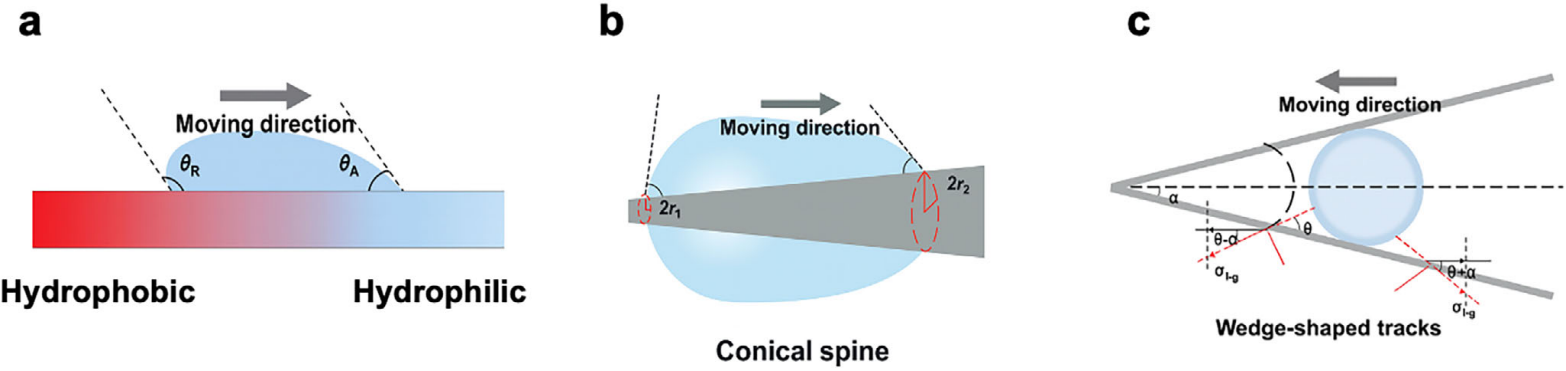
Schematic diagram of the water droplet transport behavior on the fog harvesting material on a) a hydrophobic–hydrophilic patterned surface, b) a conical spine, and c) a wedge‐shaped track, respectively. Reproduced with permission.^[^ ^35^ ^]^ Copyright 2022, Wiley‐VCH.

Surface patterned with alternating hydrophobic and hydrophilic domains, such as the back of the desert beetle, can spontaneously drive a water droplet from a hydrophobic region toward a hydrophilic zone through a surface energy gradient (**Figure** **2**a).^[^ ^40^, ^41^ ^]^ The introduction of a wettability gradient between hydrophilic and hydrophobic regions has been demonstrated to significantly enhance fog‐harvesting efficiency by coupling localized nucleation with directional droplet transport. The hydrophilic domains possess lower interfacial free energy with water, thereby serving as preferential sites for droplet nucleation due to the reduced heterogeneous nucleation energy barrier. In contrast, adjacent hydrophobic regions exhibit higher interfacial energy, discouraging condensation and maintaining a high local surface energy gradient. Once condensed droplets grow and bridge across both regions, the asymmetric contact angles generate a surface‐tension imbalance, driving spontaneous droplet motion from the hydrophobic to the hydrophilic domains.^[^ ^42^ ^]^ This is governed by the surface energy gradient force (*F*) as

(5) $$F=\gamma \int_{Lf}^{Lt}\left(cos\theta_{A}-cos\theta_{R}\right)dl$$

where γ is the surface tension of the liquid, and θ_*A*_ and θ_*R*_ are the advancing and receding contact angles of the liquid on a solid substrate, respectively. *dl* is the integration variable along the length from the hydrophobic area to the hydrophilic area.

The Laplace pressure gradient is a curvature‐induced force that moves a droplet along the structures with varying radius (Figure 2b). In nature, this is commonly observed on conical spines of cacti and tapered fibers of spider silk.^[^ ^41^ ^]^ When a droplet rests on a conical object, the two ends of the droplet have different radii of curvature (*r*_1_ at the narrower tip and *r*_2_ at the wider base). The local Laplace pressure (Δ*P*) can be expressed as

(6) $$\Delta P=2\gamma \int_{r_{1}}^{r_{2}}\frac{sin\beta }{{\left(r+R_{0}\right)}^{2}}dz$$

where *r* is the local radius of the conical spine at the two opposite sides of the droplet, β is the half‐apex angle of the conical spine, *R*_0_ is the droplet radius, and *z* is the integrating variable along the diameter of the conical spine.

Because the tip end has a higher curvature (smaller *r*_1_), its Laplace pressure is greater than that of the bottom (larger *r*_2_), creating a pressure that drives the droplet moving from the narrower side to the broader side.^[^ ^43^ ^]^ The effect is further enhanced when the cone is combined with microgrooves or surface energy gradients, as in cactus spines, which align and accelerate droplet transport toward the stem.

The liquid–gas interfacial tension gradient becomes significant when droplets are positioned on wedge‐shaped or asymmetric channels. As droplets coalesce along such a track, their shape and curvature change, creating a difference in liquid–gas interfacial tension between the two ends (Figure 2c).^[^ ^44^ ^]^ Considering a wedge‐shaped track with a half‐opening angle (α), the net force (*F*) driving droplet movement along the wedge can be expressed as

(7) $$F=2_{\sigma_{l-g}}l\left[\cos\left(\theta -a\right)-\cos\left(\theta +a\right)\right]$$

where σ_*l* − *g*_is the liquid–gas interfacial tension, and *l* is the length of the droplet contact line on one side of the wedge. Because the wedge narrows toward the tip, the curvature of the liquid–gas interface is greater on the narrower side, leading to a locally lower surface tension. This asymmetry in surface tension on the two sides of a coalesced droplet generates a net force toward the narrower end of the track to minimize the total interfacial energy, causing droplets to move spontaneously without external energy input.^[^ ^44^ ^]^ In natural systems, this principle is visible in the microchannels of Nepenthes peristomes and in certain lead vein geometries, where converging channels guide water droplets inward.

### 2.2 Bionic Fog Harvesting Surfaces Examples

#### 2.2.1 Wetability Gradient Design Inspired by The Namib Desert Beetle

**Figure 3:**
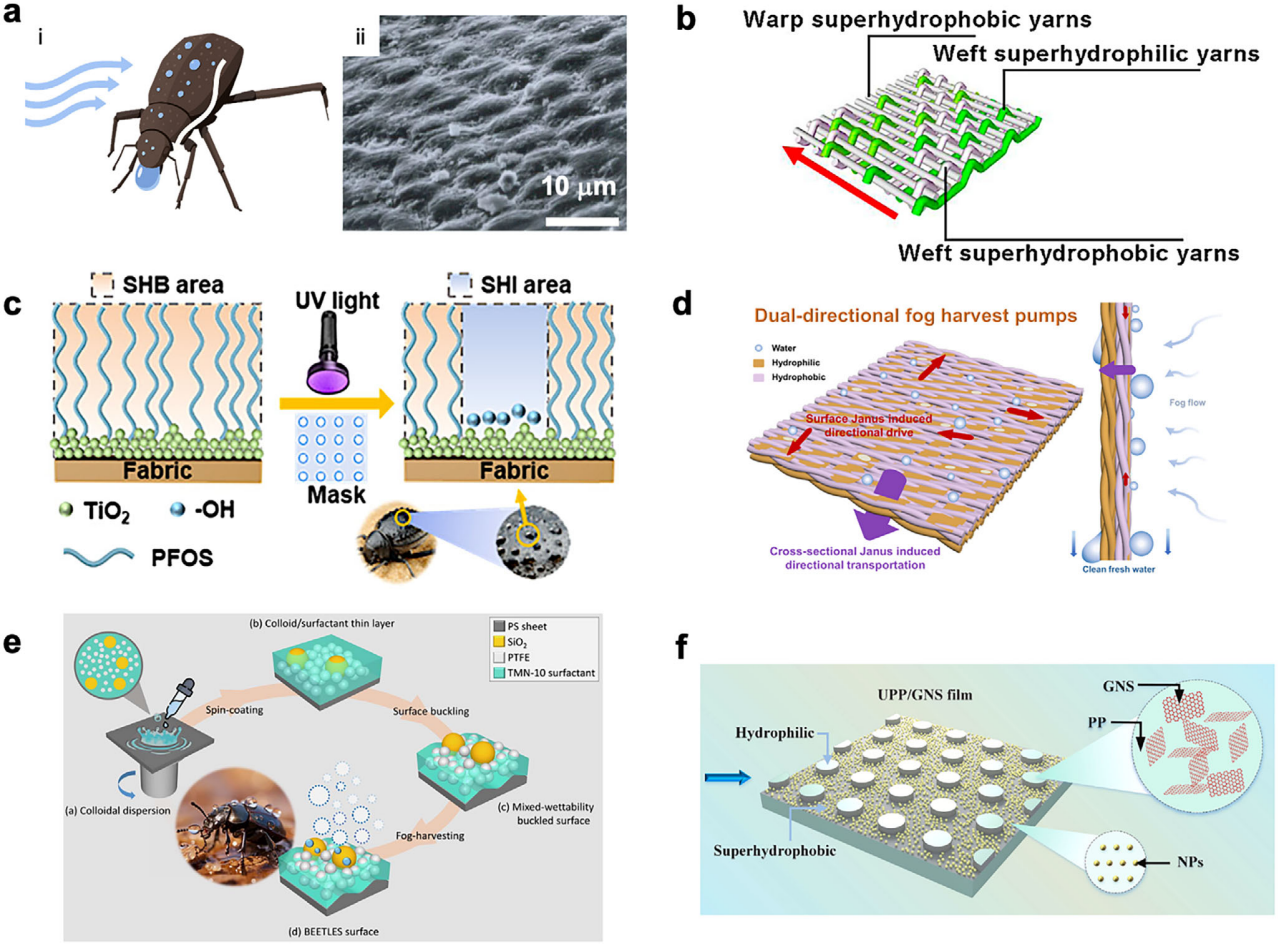
a) i) Schematic of the fog harvesting on the Namib Desert Beetle and ii) SEM image of its scale. Reproduced with permission.^[^ ^45^ ^]^ Copyright 2001, Springer Nature. b) Schematic illustration of the superhydrophobic–superhydrophilic patterned fabric. Reproduced with permission.^[^ ^47^ ^]^ Copyright 2021, ELSEVIER. c) Schematic of the UV‐irradiation‐assisted hydrophobic–hydrophilic patterning of polymer fabric. Reproduced with permission.^[^ ^48^ ^]^ Copyright 2025, American Chemical Society. d) Schematic of the beetle‐inspired Janus mesh in the cross‐sectional direction for fog harvesting. Reproduced with permission.^[^ ^49^ ^]^ Copyright 2022, American Chemical Society. e) Schematic illustration of the fabrication of desert beetle‐inspired, high‐efficiency fog‐harvesting water collectors, which consist of granular protrusions and alternating hydrophilic–hydrophobic surfaces. Reproduced with permission.^[^ ^50^ ^]^ Copyright 2025, Royal Society of Chemistry. f) Schematic of the desert beetle‐inspired united polypropylene/graphene nanosheet (UPP/GNS) fog harvesting film. Reproduced with permission.^[^ ^51^ ^]^ Copyright 2023, ELSEVIER.

The Namib Desert beetle offers one of the most cited biological blueprints for fog harvesting, owing to the unique micro‐scale surface textures on its outer shell, featuring arrays of hemispherical waxy bumps that facilitate the droplet interception and direct the water collection process (**Figure** **3**a).^[^ ^45^ ^]^ When humid air passes over the beetle's bumpy surface, the airflow is disturbed, creating micro‐vortices and promoting aerodynamically enhanced droplet collisions at the windward side of each bump, precisely where the water vapor and airborne droplets tend to accumulate and condense.^[^ ^46^ ^]^ Subsequently, the fog droplets nucleate and grow on the beetle's scale, while its hydrophobic nature minimizes droplet pinning and promotes rapid coalescence. Once droplets reach a critical size, gravity and surface tension gradients drive their efficient rolling toward the beetle's mouth. Coupling the high capture rates with continuous turnover, the beetle's surface design exemplifies an elegant strategy for sustainable fog harvesting. Inspired by the Namib Desert beetle's bumpy outer shell, the recent development of fog harvesting materials focuses on the meshed fabrics and films that are imparted with hierarchical buckles on the surfaces patterned with alternating hydrophobic and hydrophilic regions. The buckled hydrophilic region facilitates rapid nucleation and growth of the water droplet, while the hydrophobic region repels and transports the coalesced water droplets for liquid water drainage (Equations 4 and 5). Combined with the rapid heat dissipation, surface chemistry modification, Janus structures, and surface hierarchical microchannels, the engineered fog harvesting materials exhibit expeditious fog collection, directional droplet motion, and scalable manufacturability.

Among the artificial fog‐harvesting systems inspired by the Namib Desert beetle, many employ mesh‐ or fabric‐based architectures because they provide large surface areas and open structures that facilitate efficient fog interception and droplet drainage. The interconnected fibers allow continuous airflow and condensation, while their surfaces can be readily engineered with hydrophilic–hydrophobic contrasts to mimic the beetle's elytral pattern for directional water transport. Moreover, meshes are lightweight, flexible, and compatible with scalable fabrication methods such as dip coating, electrospinning, or chemical vapor deposition, making them ideal for practical, large‐area deployment in arid environments.^[^ ^22^ ^]^

Yu et al. report a patterned polyester fabric by weaving polyester yarns with alternating superhydrophobic and superhydrophilic regions, followed by in situ copper (Cu) particle deposition to enhance thermal conductivity (Figure 3b).^[^ ^47^ ^]^ The fabrication process involves pre‐treating polyester yarns to render them hydrophilic, followed by dip‐coating with polydimethylsiloxane (PDMS)/zinc oxide (ZnO) nanoparticles to achieve superhydrophobicity. The hydrophilic and superhydrophobic yarns are woven in a patterned structure to mimic the bumpy topology and wettability gradient of the beetle's back (Figure 3b). Subsequently, the deposition of Cu particles via a solution‐based redox deposition method selectively produces a thin Cu layer on the fabric. The woven hump‐like structures increase droplet–surface collision probability by enhancing the speed of air and fog droplets near the surface (thinning the boundary layer), and the copper deposition accelerates condensation dynamics by enabling efficient heat dissipation.^[^ ^47^ ^]^ As a result, this composite fabric achieves an exceptional water harvesting rate of 1432.7 mg h^−1^ cm^−^^2^, surpassing fabrics with single wettability or without copper coatings by 280 % and 30 %, respectively.^[^ ^47^ ^]^ Beyond its high performance, the technology also demonstrates advantages in durability, ultraviolet (UV) resistance, and low‐cost fabrication, highlighting the potential of bioinspired superwettable textiles as practical candidates for large‐scale fog harvesting systems.^[^ ^47^ ^]^

Li et al. develop a desert‐beetle‐inspired fog harvesting fabric by integrating titanium oxide (TiO_2_) nanoparticles and fluorosilanes (PFOS) onto polyester substrates to generate a superhydrophobic (SHB) background, followed by mask‐assisted UV irradiation to create localized superhydrophilic (SHI) domains (Figure 3c).^[^ ^48^ ^]^ The choice of UV irradiation is critical: TiO_2_ exhibits strong photocatalytic activity under UV light, decomposing PFOS modifiers and introducing surface hydroxyl groups, thereby switching wettability from SHB to SHI. By using patterned masks, this process enables precise spatial control of wettability, effectively mimicking the ideal alternating hydrophilic bumps and hydrophobic valleys for a rapid fog droplet condensation and water transport (Figure 3c). Systematic tuning of SHI pattern diameter (from 0.5 to 2 mm), SHI area proportion (2 % to 8 %), and distribution reveals the optimal balance between droplet adhesion and detachment (0.5 mm in diameter, 5 % area), leading to a maximum water collection rate of 609.1 mg·cm^−2^·h^−^1, which is 5.6 times higher than uniform SHI fabrics.^[^ ^48^ ^]^

Incorporating a Janus structure into fog‐harvesting meshes enables efficient and continuous water collection through asymmetric wettability across the thickness direction. The hydrophilic portion promotes rapid droplet nucleation and coalescence, while the hydrophobic portion facilitates droplet transport and easy release. This contrast generates a capillary pressure gradient that drives condensed water directionally, preventing flooding and maintaining active condensation sites on the harvesting surface. As a result, the mesh achieves a dynamic balance between condensation and drainage, sustaining high collection efficiency. Moreover, the tunable surface chemistry enhances durability under varying environmental conditions.^[^ ^49^ ^]^

The study by Wu et al. reports a beetle‐inspired Janus mesh for fog harvesting (Figure 3d).^[^ ^49^ ^]^ The two sides of the mesh have two distinct wettability (e.g., hydrophilic vs. hydrophobic), enabling directional liquid transport across the fabric by Janus wetting.^[^ ^49^ ^]^ The advantage of this Janus structure compared to conventional fog‐harvesting fabrics lies in its synergistic dual wettability: in addition to the purely hydrophilic–hydrophobic patterned fabric surface that captures fog and transports water droplets on the surface, the cross‐sectional Janus (CS‐Janus) design promotes water transport in across the fabric, thus prevents water accumulation that would otherwise block active sites, reduces evaporation losses, and sustains continuous harvesting.^[^ ^49^ ^]^ Specifically, the material is constructed on stainless steel mesh decorated with vertically aligned ZnO nanorods to render superhydrophilicity, followed by a one‐sided spray‐and‐dry deposition of stearic acid that converted into zinc stearate, forming a stable superhydrophobic layer.^[^ ^49^ ^]^ This asymmetric design creates both a beetle‐like surface structure and a CS‐Janus structure (Figure 3d). During fog collection, the hydrophilic sites enhance nucleation and capture of tiny droplets, while the hydrophobic regions and asymmetric wettability facilitate droplet coalescence, directional transport, and timely removal across the mesh. The optimized Janus mesh achieves a high‐water collection rate of 2478.73 mg m^−2^ h^−1^, outperforming homogeneous hydrophilic by up to 2.6 times.^[^ ^49^ ^]^ Beyond efficiency, the mesh shows durability against abrasion, antibacterial activity, and stable long‐term water collection.^[^ ^49^ ^]^

Despite the advantages of woven fabrics and meshes, such as the beetle‐inspired polyester–copper composites in scalability, flexibility, and low cost, there are also potential disadvantages that need to be considered. First, weaving mainly provides millimeter‐scale to sub‐millimeter features. Unlike lithography or electrospinning, it is difficult to control fine micro‐/nanostructures that can enhance nucleation density and surface energy gradients. This may cap water‐harvesting performance compared to more finely engineered surfaces.^[^ ^22^ ^]^ Second, superhydrophilic yarns can absorb water into the fabric matrix rather than transporting it outward, which reduces the efficiency of droplet release and collection.^[^ ^22^ ^]^ This “wicking” effect could compete with the desired directional transport.

To remedy the aforementioned shortcomings intrinsic to meshes and fabrics as fog harvesting materials, Xu et al. report fabrication of hierarchical buckled microchannels on heat‐shrinkable polystyrene (PS) substrates with alternating hydrophilic–hydrophobic domains (Figure 3e).^[^ ^50^ ^]^ Hydrophilic silica oxide (SiO_2_) particles, as well as alternatives such as calcium carbonate (CaCO_3_), ZnO, and diatomaceous earth, are dispersed together with hydrophobic polytetrafluoroethylene (PTFE) micelles and deposited onto the pre‐treated PS sheets by spin coating, followed by thermal shrinkage to induce spontaneous buckling. Here, the hydrophilic protrusions promote condensation, while hydrophobic ridges act as microchannels for droplet coalescence and directional transport (Figure 3e). Unlike the previous beetle‐inspired designs that rely on fabrics or meshes, which are prone to clogging issues, this work enables uniform surface coverage on a solid sheet and greatly simplifies scale‐up. The optimized configuration, with 10 µm SiO_2_ at a 1:300 SiO_2_:PTFE ratio, enhances fog harvesting to 1069.0 mg cm^−2^ h^−1^ by aligning the microbuckles parallel to droplet flow.^[^ ^50^ ^]^ It is worth noting that integration of the beetle‐inspired microscale patterning with bird's nest fern‐inspired funnel‐shaped macroscopic geometry promotes effective large‐scale water collection, harvesting ≈125 g overnight in field tests (for a sample size of 15 cm in length and 4.5 cm in width).^[^ ^50^ ^]^ Compared to flat or single‐wettability surfaces, the alternating wettability combined with buckled channels improves water capture by ≈30%.^[^ ^50^ ^]^

Micropillars, mimicking the bumpy beetle's scale, can provide abundant nucleation sites on the hydrophilic tops for rapid droplet condensation, while the hydrophobic valleys reduce adhesion and enable coalescence‐induced jumping. The hierarchical topography establishes a hybrid wettability and also generates a capillary gradient that drives directional droplet transport from the valleys to the pillar tops and then downward under gravity, maintaining continuous surface renewal. Zhu et al. design a united polypropylene/graphene nanosheet (UPP/GNS) fog harvesting film that combines melt processing and molding with post‐surface modification (Figure 3f).^[^ ^51^ ^]^ The fabrication begins with melt blending polypropylene (95 wt.%) and graphene nanosheets (5 wt.%) at 210 °C, producing a mechanically durable and photothermally active composite. This melt is extruded and compression molded into 40 × 40 × 2 mm^3^ films with micro‐pillar arrays templated by porous plates of controlled pore size (0.5–1.5 mm). The structured surfaces are then spray‐coated with a superhydrophobic mixture containing PDMS, silica nanoparticles, and hexamethyl disilylamine. To engineer hybrid wettability, the films are abraded against sandpaper so that the superhydrophobic coating is selectively removed from the tops of the pillars, leaving them hydrophilic while retaining superhydrophobicity on the sidewalls and base.^[^ ^51^ ^]^ This fabrication strategy is advantageous in several ways. First, the use of extrusion compression molding enables rapid, continuous, and industrially scalable production compared with lithography‐ or etching‐based approaches commonly used in bioinspired surfaces. Second, the selective removal process to achieve hydrophilic tops and superhydrophobic sides is both simple and effective, which creates a functional analog of the desert beetle's back that promotes condensation, droplet coalescence, and directional transfer. Third, the integration of graphene nanosheets not only enhances structural durability but also imparts photothermal responsiveness, enabling active anti‐icing performance in addition to passive freezing delay. Collectively, the UPP/GNS film achieves a record fog collection of 1251 mg cm^−2^ h^−1^ while maintaining chemical tolerance, durability, and environmental adaptability.^[^ ^51^ ^]^

#### 2.2.2 Laplace Pressure Gradient Design Inspired by The Cacti Spines

**Figure 4:**
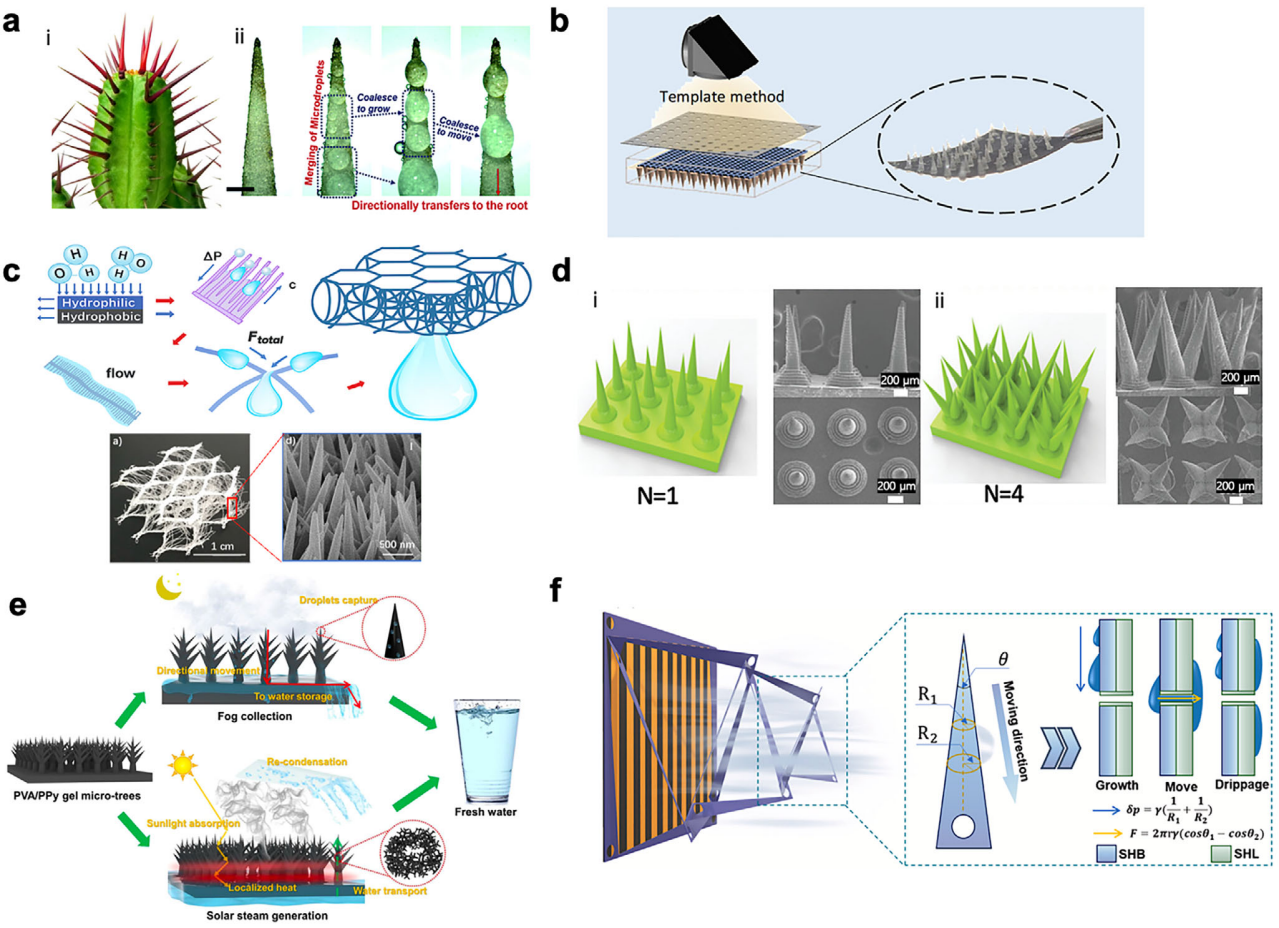
a) Schematic of i) the cactus spine and ii) the conical‐shape spine water harvesting as a result of Laplace pressure gradient. Scale bar: 1 mm. Reproduced with permission.^[^ ^27^ ^]^ Copyright 2020, Royal Society of Chemistry. b) Schematic of the cactus‐inspired superwetting mesh fabricated by integrating a template method with a photocuring process. Reproduced with permission.^[^ ^52^ ^]^ Copyright 2024, American Chemical Society. c) Illustration of the structure and the water‐collecting mechanism of bioinspired N3D. Inset: photo of the N3D (left) and SEM image of the nanocones decorated on the N3D (right). Reproduced with permission.^[^ ^53^ ^]^ Copyright 2019, American Chemical Society. d) Photo and SEM images of the 3D‐printed cactus‐mimetic array with different number of spines: i) N = 1 and ii) N = 4. Reproduced with permission.^[^ ^55^ ^]^ Copyright 2020, WILEY‐VCH. e) Conceptual representation of the PVA/PPy hydrogel membrane with micro‐topologies that is capable of 24‐h freshwater harvesting. Reproduced with permission.^[^ ^57^ ^]^ Copyright 2021, Springer Nature. f) Schematic mechanism of the fog capture and droplet transport on the 3D origami cage structure. Reproduced with permission.^[^ ^60^ ^]^ Copyright 2025, ELSEVIER.

The cactus provides another compelling natural model for fog harvesting, with its spines acting as multifunctional structures that integrate droplet capture, condensation, and transport.^[^ ^27^ ^]^ The cactus spines can efficiently harvest fog due to their conical geometry and microgrooved texture, which together create a self‐driven pathway for condensed water to move toward the plant body. When humid air flows across the spine, the sharp tip with its small radius of curvature acts as a preferential site for heterogeneous nucleation, because it locally amplifies the surface energy and vapor concentration gradients, thereby reducing the energy barrier for droplet formation. The high curvature at the tip enhances molecular attraction to polar water vapor, while the altered airflow forms a slight stagnation zone that increases local vapor residence time, making condensation more likely to occur there than on flatter regions.^[^ ^26^ ^]^ As droplets form and coalesce, a Laplace pressure gradient develops along the conical spine: according to Equation 6, the higher curvature (*r*_1_ of 2–5 µm) at the tip produces greater internal pressure than the lower curvature (*r*_2_ of 20–60 µm) near the base, driving water droplets spontaneously from the tip toward the base (**Figure** **4**a). The gradual tapering of the spine optimizes this curvature‐induced pressure gradient, ensuring a continuous driving force that promotes unidirectional droplet transport. In addition, directional grooves and asymmetric barbs act as capillary tracks, and subtle wettability variations, e.g., hydrophilic near the tip and slightly hydrophobic near the base, further bias droplet motion. Together, these multiscaled structural and chemical features maximize condensation efficiency and exploit the optimized Laplace pressure gradient for continuous, passive fog collection and water delivery to the cactus stem.^[^ ^27^ ^]^ This design not only maximizes water capture efficiency in arid environments by continuously renewing condensation sites at the apex but also ensures reliable delivery of harvested droplets to the plant body for storage. By coupling localized nucleation with passive directional transport, the cactus spine architecture exemplifies a robust and scalable strategy for atmospheric water harvesting, inspiring engineered materials that exploit geometry‐driven Laplace pressure gradients, anisotropic wetting, and bio‐mimetic surface texturing.

Deng et al. report a cactus‐inspired superwetting mesh fabricated by integrating a template method with a photocuring process, in which PDMS molds containing conical cavities are filled with trimethylolpropane ethoxylate triacrylate (ETPTA) and cured under xenon lamp irradiation to grow microneedles firmly on copper meshes (Figure 4b).^[^ ^52^ ^]^ Surface wettability is tuned via octadecanethiol modification, enabling the preparation of either superhydrophilic copper mesh with superhydrophilic needles (HICM‐NA) or superhydrophobic copper mesh with hydrophilic needles (HOCM‐NA). The HICM‐NA exhibits a biomimetic mechanism akin to cactus spines: the conical geometry and groove structures generated Laplace pressure and enhanced capillary forces, while the gradual deepening of grooves from tip to base amplified directional liquid transport. Furthermore, liquid film formation along the microneedles reduces frictional resistance and promotes continuous droplet pumping toward the mesh, thereby accelerating coalescence and drainage. The cactus‐inspired architecture achieves fog harvesting performance, with HICM‐NA, up to 17.7 kg h^−1^ m^−2^, while the meshes without a conical needle array only collect 7.6 kg h^−1^ m^−2^ (HIMC).^[^ ^52^ ^]^ This design combines scalable fabrication, tunable wettability, and efficient droplet management, offering clear advantages in both capture efficiency and transport speed over flat or purely hydrophobic/hydrophilic surfaces.

To evolve from a 1D substrate to a 3D fiber network for fog harvesting, Li et al. develop a nanocone‐decorated 3D fiber network (N3D) where ZnO nanocones are uniformly grown on a lightweight multi‐intersection nylon web (Figure 4c).^[^ ^53^ ^]^ The fabrication involves pretreating the nylon web, depositing a ZnO nanoparticle seed layer through repeated soaking in zinc acetate/sodium hydroxide colloidal solution and thermal treatment, followed by hydrothermal growth in a zinc nitrate/hexamethylenetetramine aqueous solution at 95 °C for 4 h in a Teflon reactor, yielding dense and durable nanocones of 2–3 µm height and 200–300 nm diameter (Figure 4c). This multiscale integration provides an exceptionally high surface area for nucleation, while the 3D network architecture promotes efficient droplet coalescence and transport.^[^ ^53^ ^]^ This transformation enabled continuous water flow with drastically reduced retention forces. The combination of nanoscale conical decoration with a 3D fibrous scaffold contributes to outstanding fog‐harvesting efficiencies up to 865 kg m^−2^ day^−1^, more than 240 times the weight of the web collected in just 2 h.^[^ ^53^ ^]^

3D printing offers a precise and versatile approach for fabricating cactus‐inspired fog‐harvesting materials. It enables accurate replication of the cactus spine's conical geometry and surface grooves, allowing fine control over the Laplace pressure gradient that drives directional water transport. The technique also permits the design of hierarchical textures and tunable wettability gradients to enhance droplet nucleation and coalescence. With its flexibility in material selection and rapid prototyping, 3D printing facilitates systematic optimization of structure and performance.^[^ ^54^ ^]^ Li et al. fabricate a natural cactus‐inspired multibranched cluster spines material using an immersed surface accumulation–based 3D printing (ISA‐3DP) technique, which enables the precise reproduction of multibranched spine clusters that are difficult to achieve through traditional methods such as etching or replica molding (Figure 4d).^[^ ^55^ ^]^ The spines are printed from a photocurable nanocomposite resin of E‐glass and multiwalled carbon nanotubes (MWCNTs), and their surface wettability is optimized by applying a nanoscale hydrophobic coating. The fog collection mechanism arises from synergistic effects: water condensation at the sharp spine tips due to high curvature, reduction of the critical nucleation radius on hydrophobic surfaces, and directional droplet transport driven by Laplace pressure gradients along the conical geometry. Additionally, multibranched and hexagonally arranged spines enhance turbulent airflow, increase interception of fog droplets, and promote continuous coalescence and transport, which ensures rapid regeneration of condensation sites (Figure 4d). Performance tests show that a single spine with an optimized tip angle of 10° and hydrophobic coating achieved a maximum water collection rate of 2 mg min^−1^ mm^−3^, while multibranched clusters collected up to four times more water than that of the single spine.^[^ ^55^ ^]^ Compared to earlier cactus‐inspired materials that are limited to single spines or simple arrays,^[^ ^52^, ^53^ ^]^ this work leverages advanced 3D printing to construct customizable multibranched architectures with optimized arrangements and wettability control, thereby significantly enhancing fog harvesting efficiency and offering a scalable pathway toward next generation, energy‐free water collection surfaces.

Hydrogel, as a 3D crosslinked polymer network that combines water affinity, storage ability, and tunable surface chemistry, can be used for moisture regulation and liquid water retention.^[^ ^1^ ^]^ Shi et al. introduce a poly(vinyl alcohol)/polypyrrole (PVA/PPy) hydrogel membrane engineered with three‐dimensional micro‐tree arrays that mimic the conical geometry of cactus spines. Fabrication is achieved through computer‐aided design (CAD), stereolithography 3D printing, and a double‐inverse molding process, enabling precise construction of 4 mm‐tall branched micro‐cones with tip sizes down to ≈20 µm (Figure 4e).^[^ ^57^ ^]^ The hierarchical conical structures generate Laplace pressure gradients that drive directional droplet transport, allowing efficient fog capture while preventing surface clogging. Additionally, the porous hydrogel matrix enhances water transport, reduces evaporation enthalpy, and incorporates PPy for light absorption, thereby supporting high‐performance solar steam generation (Figure 4e). This diurnal mechanism—fog collection at night and solar‐driven evaporation during the day—makes the system a true 24‐h water harvester. The material demonstrates significant advantages over conventional meshes and even natural cactus stems, with fog collection rates of ≈5.0 g cm^−2^ h^−1^ and a solar vapor generation rate of 3.64 kg m^−2^ h^−1^ under 1 sun. Outdoor tests show daily water yields of ≈34 L m^−2^, coupled with long‐term durability over 20 months, highlighting the synergy of bioinspired structure and hydrogel functionality for scalable and sustainable fog harvesting.^[^ ^57^ ^]^

Kirigami and origami, the oriental paper arts, can create intriguing geometrical structures through cutting and folding of paper. The folded structures enhance fog harvesting by combining increased surface area, multidirectional exposure, and tunable geometry.^[^ ^58^, ^59^ ^]^ The cactus‐inspired origami fog‐harvesting material, developed by Chen et al., integrates bio‐inspiration with structural engineering to maximize fog capture.^[^ ^60^ ^]^ Copper sheets are first chemically etched and thermally treated to form superhydrophilic copper oxide (CuO) “peony flower” lamellae, then modified with n‐dodecanethiol to create superhydrophobic copper sulfide (CuS) papillae, and finally subjected to laser etching to achieve Janus wettability (Figure 4f). This origami lattice design mimics the conical spines of cactus for Laplace‐pressure‐driven droplet transport and the lotus‐leaf papillae for rapid droplet shedding, while the foldable origami architecture enables transformation from 2D to 3D for portable deployment and storage.^[^ ^60^ ^]^ The hierarchical wettability and cage‐like geometry establish a three‐stage capture mechanism: primary fog interception on superhydrophobic sites, secondary collision‐driven trapping inside the cage, and tertiary migration to superhydrophilic domains for drainage. Advantages of this system include its collapsibility, high cyclic stability, anti‐icing behavior, and compatibility with urban agriculture through direct irrigation. Performance tests demonstrate a remarkable 463.45% increase in fog collection efficiency over untreated copper sheets of equal area without any modification, underscoring the synergy between cactus‐inspired conical transport, origami‐enabled multi‐capture, and Janus surface chemistry in advancing sustainable water harvesting technologies.^[^ ^60^ ^]^

#### 2.2.3 Dual Biomimetic Design (Wettability and Laplace pressure) Inspired by the Spider Silk

**Figure 5:**
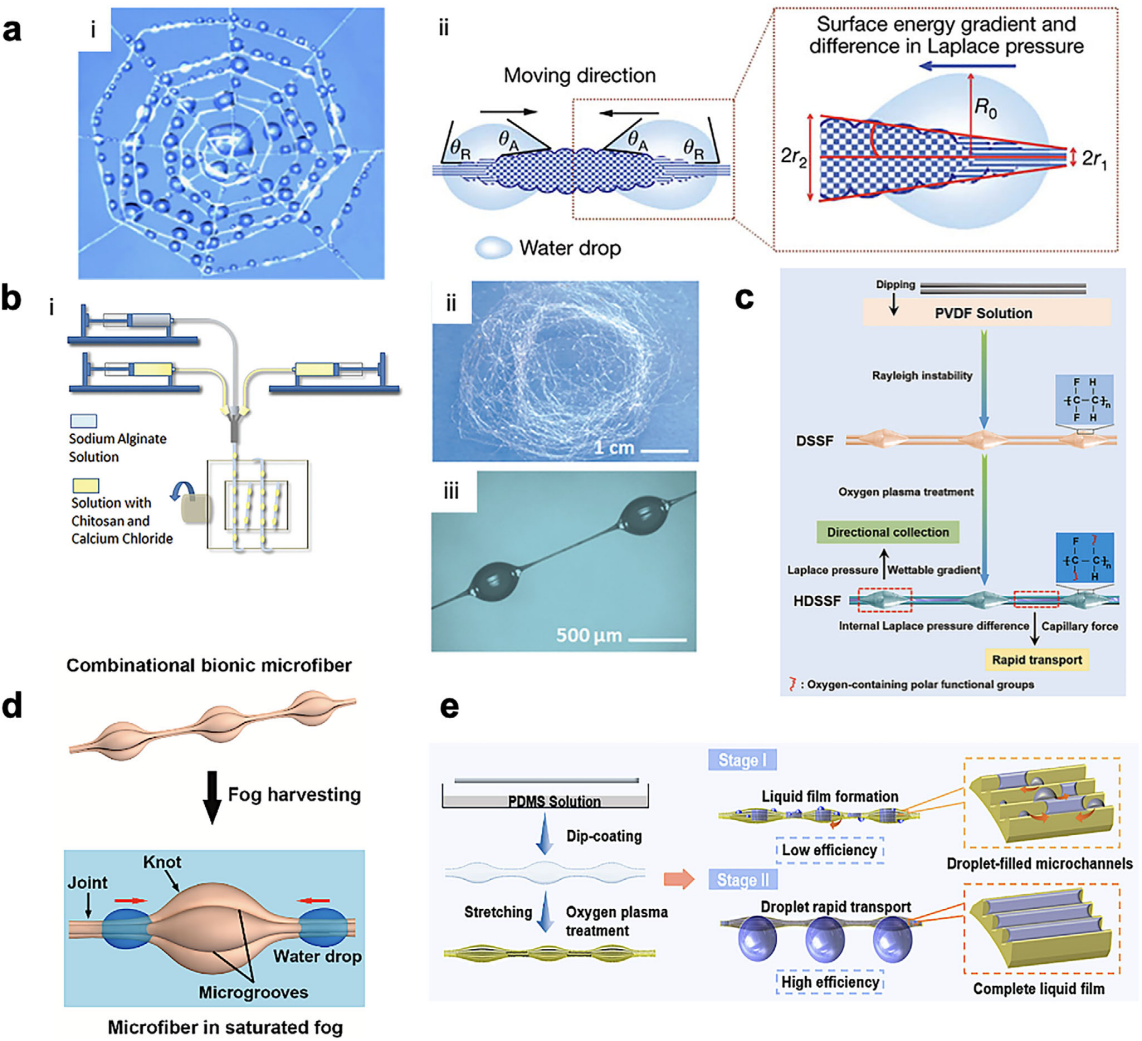
a) Schematic of i) the spiderweb fog harvesting, reproduced with permission.^[^ ^66^ ^]^ Copyright 2020 American Chemical Society. and ii) the illustration of water harvesting mechanisms of a spider silk fiber by the surface energy gradient and Laplace pressure. Reproduced with permission.^[^ ^61^ ^]^ Copyright 2010, Springer Nature. b) i) Schematic illustration of fabrication of bioinspired microfiber via microfluidic method, ii) optical image of the collected microfiber in large quantity, iii) microscopic image of the microfiber with spindle knots. Reproduced with permission.^[^ ^62^ ^]^ Copyright 2020, WILEY‐VCH. c) Schematic illustration of the fabrication process of the HDSSF. Reproduced with permission.^[^ ^63^ ^]^ Copyright 2023, ELSEVIER. d) Schematic of the combinational biomimetic multithread microfiber (CBM) with knot‐joint microstructure inspired by spider silk and surface microgrooves for efficient fog harvesting. Reproduced with permission.^[^ ^64^ ^]^ Copyright 2022, ELSEVIER. e) Schematic illustration of the fabrication process of the HMSSF and droplet movement on the HMSSF. After the liquid film is formed, captured droplets collected on the HMSSF could be rapidly transported toward the center of the knot due to the difference between the capillary force and the Laplace pressure. Reproduced with permission.^[^ ^65^ ^]^ Copyright 2025, American Chemical Society.

Spider silk can harvest fog through a synergistic combination of surface chemistry, micro–structural geometry, and capillary‐driven water transport mechanisms that together enable efficient droplet nucleation, growth, coalescence, and directional movement. The fog‐harvesting mechanism of spider silk arises from its unique “wet‐rebuilt” fiber architecture. When exposed to humid air, the cribellate spider silk undergoes a structural transformation: fluffy puffs of nanofibrils shrink into periodic spindle‐knots connected by slender joints. The thicker spindle‐knots, composed of randomly oriented nanofibrils, are highly hydrophilic and act as preferential condensation and collection sites, while the thinner and slightly more hydrophobic joints, made of aligned nanofibrils, primarily serve as initial nucleation and transport sites for water droplets (**Figure** **5**a). This periodic heterogeneity in both surface energy and curvature is crucial for fog harvesting. When airborne microdroplets collide with or condense on the fiber, heterogeneous nucleation preferentially occurs on the spindle knots’ joints due to their lower nucleation energy barrier. Once fog is condensed, droplets on the joints spontaneously migrate toward the spindle‐knots, driven cooperatively by two key forces: a surface energy gradient caused by differences in nanoscale roughness and wettability (Equation 5), and a Laplace pressure difference generated by the thinner joint and the thicker spindle‐shaped geometry of the knots (Equation 6 and Figure 5a).^[^ ^61^ ^]^ This dual driving mechanism overcomes contact line hysteresis that typically immobilizes micrometre‐sized droplets, enabling continuous directional transport and coalescence into larger drops at the spindle‐knots.^[^ ^61^ ^]^ Ultimately, the spider silk's hierarchical design allows for a cycle of preferential nucleation, rapid coalescence, directional motion, and gravitational shedding, effectively converting dispersed airborne vapor into liquid water. These natural principles have inspired the development of bioinspired synthetic fibers that not only replicate the spindle–joint architecture but also impart microchannels on the fibers for highly efficient passive and scalable fog‐harvesting systems in arid and coastal regions.

Liu et al. report a spider silk–inspired fiber fabricated using a parallel‐nozzle microfluidic technique, in which sodium alginate solution is pumped through the central channel, while chitosan solution containing calcium chloride is simultaneously pumped through two side channels (Figure 5b).^[^ ^62^ ^]^ Upon contact, calcium (Ca) ions rapidly crosslink the alginate into a continuous gel fiber, while the chitosan coats the surface, through a surface‐tension‐driven process in which continuous liquid films are inherently unstable and tend to break up into droplets (Rayleigh instability), breaking into discrete droplets that form the characteristic spindle‐knots along the fiber axis (Figure 5b). By adjusting the relative flow rates of the alginate and chitosan solutions, the researchers can finely control the spacing, width, and height of the spindle knots, enabling systematic tuning of the fiber morphology. The combination of chitosan (polycation) and alginate (polyanion) provides strong electrostatic interactions that stabilize the heterogeneous spindle‐knot/joint structure, while the incorporation of calcium chloride further modulates knot roughness and curvature.^[^ ^62^ ^]^ These mix‐structured microfibers exhibit gradients in both curvature and surface roughness, which generate Laplace pressure and surface energy differences that drive captured droplets to coalesce and move directionally toward the knots, effectively mimicking natural spider silk. In fog harvesting tests, optimized fibers achieve water collection up to 4.47 g h^−1^ per a single fiber, with parallel arrays of six fibers reaching 24.5 g h^−1^ and assembled artificial webs harvesting 600 g h^−1^, underscoring their promise for large‐scale water harvesting applications.^[^ ^62^ ^]^

However, in the conventional single‐thread bioinspired spider silk fiber (BSSF) design, the available surface area for droplet nucleation is limited, and droplet coalescence can cause partial blockage or backward flow, reducing the overall water collection efficiency. By contrast, the dual‐ and multi‐thread designs can introduce capillary bridging between adjacent threads, accelerating droplet coalescence and transport along the fiber pair. Additionally, they offer a greater effective surface area. Huan et al. developed a dual‐thread spider silk fiber by fixing two nylon fibers (≈20 µm diameter) side by side on a U‐shaped holder and dip‐coating them into polyvinylidene fluoride/dimethylformamide (PVDF/DMF) solutions of controlled concentrations.^[^ ^63^ ^]^ Upon withdrawal at specific pulling velocities, Rayleigh instability drives the PVDF film to spontaneously break up into periodic spindle knots along the fibers, mimicking the natural spider silk. After drying, the fibers underwent oxygen plasma treatment, which introduced oxygen‐containing polar groups and greatly improved surface hydrophilicity (Figure 5c).^[^ ^63^ ^]^ The dual‐thread configuration plays a critical role: by introducing two closely aligned threads, a narrow microchannel is formed between them, which can generate strong capillary forces, enabling a rapid suction of the condensed microdroplets into a continuous liquid film. This film then connects adjacent spindle knots, ensuring that droplets are rapidly transported and coalesced at the knots under the combined effect of capillary forces and Laplace pressure differences (Figure 5c).^[^ ^63^ ^]^ Compared with conventional BSSFs, which suffer from slow droplet transport and in situ growth on threads, the dual‐thread design significantly accelerates surface regeneration and maintains stable fog harvesting cycles. This structural improvement boosts the water collection rate to 9.03 g cm^−2^·h^−1^, ≈590% higher than that from normal BSSFs, highlighting the importance of integrating dual‐thread microchannels for efficient and sustainable fog harvesting.^[^ ^63^ ^]^

To further enhance the water collection efficiency from the dual‐thread spider silk fibers, the multi‐thread spider silk‐mimicking fibers are fabricated from sodium alginate and polyvinyl alcohol precursor solutions, which are ionically crosslinked in a calcium chloride bath using a dynamic interfacial spinning (DIS) process (Figure 5d).^[^ ^64^ ^]^ By imposing a programmable vertical vibration on stainless steel nozzles positioned at the air–liquid interface, this method allows precise formation of periodic spindle knots and slender joints characteristic of natural spider (Figure 5d). Importantly, multi‐axial nozzle assemblies are also developed, enabling the simultaneous extrusion of two or four parallel streams that bind during dehydration to form dual‐ or multi‐thread fibers (diameter less than 20 µm per thread) with cactus‐spine‐like surface grooves. Compared to the single‐thread spider‐silk‐mimicking fibers, the dual‐ and quadri‐thread combinational biomimetic microfibers (CBMs) (>100 µm in diameter) demonstrate an additional enhancement due to groove‐assisted capillary driving forces. Specifically, quadri‐thread CBMs collect ≈0.400 ± 0.027 µL of water on a single knot within 24 s, which is ≈1.76 times more than single‐thread single knot under identical fog conditions.^[^ ^64^ ^]^ Nevertheless, this method suffers from a major drawback: captured droplets are largely retained near the knots and grow in situ, and only a small fraction can be transported to the center by Laplace pressure.^[^ ^65^ ^]^ This inefficient droplet transport can slow the water collection process and limit overall harvesting efficiency. To overcome this, Wang et al. dip‐coat elastic polyurethane filaments with PDMS to generate spindle‐knot structures via Rayleigh instability, followed by mechanical stretching to induce uniform surface wrinkling and microchannel formation (Figure 5e).^[^ ^65^ ^]^ Subsequent oxygen plasma treatment rendered the surface hydrophilic. Here, microchannels are introduced across the entire fiber surface simply by adjusting the stretching rate, and their depth and density can be finely controlled without the need for complex dual‐thread assembly or precise alignment in earlier designs. Mechanistically, the spindle knots act as fog collection centers, while the numerous axial microchannels mimic Sarracenia trichomes, enabling rapid droplet transport through combined capillary force and Laplace pressure (Figure 5e). This dual‐stage collection process, initial microchannel filling followed by liquid‐film‐assisted rapid transport, significantly reduces droplet detachment time and enhances water transfer efficiency. Compared with conventional single‐thread fibers, the hydrophilic microchannel fibers demonstrate a fog collection rate of 0.34 µL s^−1^, representing a 467 % improvement, with stable performance over 30 cycles.^[^ ^65^ ^]^

#### 2.2.4 Ternary Biomimetic Design (Wettability, Laplace Pressure, and Interfacial Tension) Inspired by The Nepenthes Alata

**Figure 6:**
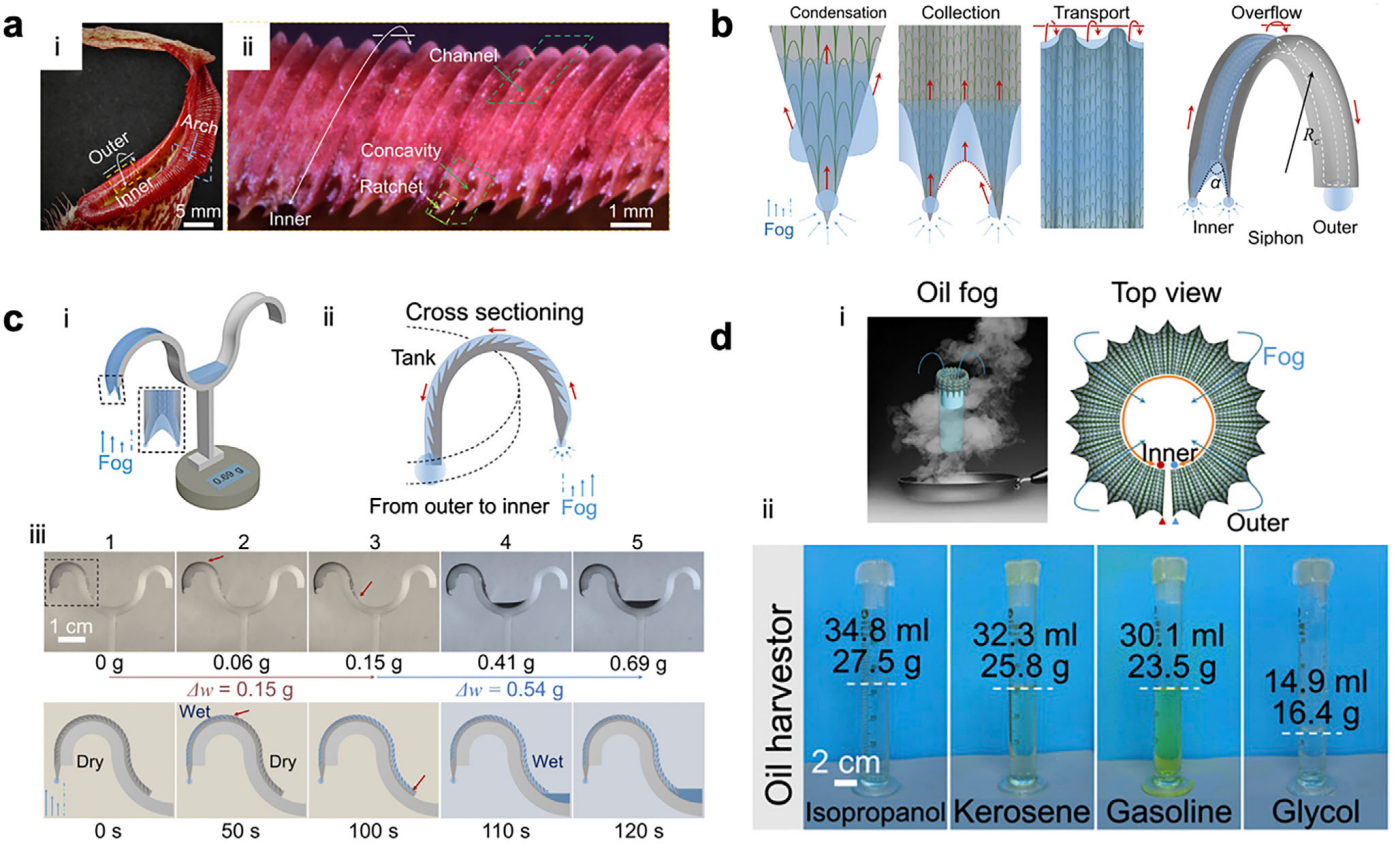
a) i) Optical image of the Nepenthes alata pitcher, ii) inner view of the ratchet teeth projection along the inner edge of the peristome. b) Schematic images of water condensation and transport on the peristome. Water condenses on the ratchet teeth first and then transports upward to the concavity, which acts as both a collector and a pump to deliver water until it overflows the arch‐shaped channel to the outer rim. The communicating vessels constructed after wetting speed up the water‐harvesting efficiency. c) i) Schematic diagram of the artificial peristome fog harvester, ii) schematic of the condensate water can be transported from the atmosphere to the inner tank of the harvester, iii) time‐sequence images of the water condensation and transport process on the artificial peristome harvester. d) i) Schematic of the oil fog harvesting, ii) pictures of the oil fog from isopropanol, Kerosene, Gasoline, and glycol (left to right) gathered and stored in the artificial peristome harvester. Reproduced with permission.^[^ ^31^ ^]^ Copyright 2020, PNAS.

Nepenthes alata, a tropical pitcher plant, harvests fog water through its hydrophilic, ridge‐patterned peristome surface (**Figure** **6**a).^[^ ^31^ ^]^ The fog‐harvesting capability of Nepenthes alata arises from the synergistic coupling of wettability, Laplace pressure, and liquid–gas interfacial tension gradients along its hierarchically structured peristome surface for efficient directional collection and transport of water from airborne fog droplets to the pitcher interior.^[^ ^67^ ^]^ The peristome features radially arranged wedge‐shaped microgrooves that exhibit a wettability gradient (Figure 6b), from less hydrophilic outer rims to highly hydrophilic inner regions, creating a surface energy gradient that drives spontaneous inward spreading of condensed droplets.^[^ ^31^ ^]^ Upon fog exposure, heterogeneous nucleation occurs preferentially within the hydrophilic grooves, after which the coalesced droplets experience a Laplace pressure gradient induced by curvature differences between the convex ridge tips and concave groove interiors (Figure 6b). This pressure imbalance generates a force that continuously transports water from regions of higher curvature (the outer edge) toward regions of lower curvature (inner peristome). In Nepenthes alata, each radial groove of the peristome acts as a wedge‐shaped capillary track, which is narrower at the inner region and wider toward the outer rim. As condensation, coalescence, and local evaporation proceed, the water droplets bridge the two sides of the wedges; thus, the asymmetric curvature of the liquid–gas interface of the droplet naturally leads to an unbalanced tension gradient, which arises along the meniscus and continuously propels the droplet inward to the narrower side to minimize interfacial energy (Equation 7). Combined with the slippery wetting state of the peristome and the influence of gravity, this synergy ensures that the collected water is funneled directly into the pitcher cavity continuously. Meanwhile, the continuous removal of water opens up fresh nucleation sites, sustaining a cyclic, lossless water‐harvesting process from airborne moisture – mechanisms that have inspired the design of bioinspired fog‐harvesting surfaces with hierarchical grooves and wedge‐shaped tracks.^[^ ^31^ ^]^

Li et al. integrate multi‐scaled curvatures, ratchet teeth, wedge concavities, and arch channels to achieve continuous and ultrafast fog harvesting and liquid transport (Figure 6c).^[^ ^31^ ^]^ The artificial structures are fabricated using digital light 3D printing, which enables micrometer‐scale precision and customizable replication of the complex hierarchical curvatures, followed by replication with hydrophilic PVA hydrogel for fog harvesting. The mechanism relies on synergistic Laplace pressure gradients and surface energy release from the droplets at the ratchet–concavity interface, which collectively drive directional, antigravity liquid motion and form a self‐sustained slippery aqueous layer that further accelerates transport (Figure 6c). The fog‐harvesting performance of the Nepenthes alata–inspired artificial peristome is quantified using the hydrogel replica mounted on a glass cylinder (Figure 6c). There is 40.1 mL of water within 2 h at room temperature, corresponding to an average collection rate of ≈6.1 × 10^−3^ g cm^−2^·s^−1^, which is 20‐fold higher water collection efficiency compared to its dry state.^[^ ^31^ ^]^ Furthermore, Li et al. demonstrate harvesting of organic vapors. Using a PDMS oleogel replica, the device collects organic fogs at high and stable rates over long‐term operation: ≈4.2 × 10^−3^ g cm^−2^·s^−1^ for isopropanol, 3.7 × 10^−3^ g cm^−2^·s^−1^ for kerosene, 3.4 × 10^−3^ g cm^−2^·s^−1^ for gasoline, and 2.5 × 10^−3^ g cm^−2^·s^−1^ for glycol, with performance maintained for up to 120 h (Figure 6d).^[^ ^31^ ^]^ This dual capability arises from the ability to tune surface chemistry through material choice—hydrophilic hydrogel to attract water and oleophilic PDMS to capture organic vapors, while the structural curvatures universally guide condensate motion. Such a synergy between multiscale geometry and surface chemistry endows the system with exceptional versatility, making it a promising candidate for scalable applications in atmospheric water collection and industrial vapor recovery.

Building on the nature‐inspired strategies demonstrated in fog harvesting systems, such as the hydrophilic–hydrophobic patterning of the desert beetle's scale, the conical Laplace pressure gradient of cactus spines, the periodic curvature and wettability modulation of spider silk, and the slippery directional tracks of Nepenthes alata, significant insights have been gained into efficient droplet nucleation, coalescence, and directional transport under high‐humidity environments. Representative examples, along with their inspirations, fabrication methods, structural features, and performances, are summarized in **Table** **1**. However, fog harvesting inherently depends on the presence of micrometer‐sized droplets suspended in the air, limiting its applicability in arid or low‐humidity regions where liquid water is scarce. To overcome this limitation, recent research has also shifted toward sorption‐based water vapor harvesting, which is the direct condensation or sorption of molecular water vapor from unsaturated air. This next‐generation approach draws upon similar interfacial principles of topology, capillarity, and surface energy manipulation, while incorporating sorptive and thermoresponsive materials, e.g., hygroscopic salts, metal–organic frameworks (MOFs), and hydrogels, capable of capturing water vapor at the molecular scale. Thus, the design evolution from fog to vapor harvesting represents a continuum of nature‐inspired strategies aimed at achieving all‐weather, energy‐efficient atmospheric water collection.

**Table 1:** Summary of recent fog‐harvesting systems with bioinspired design strategies.

Design basis	Inspiration	Method	Structure/Scale	Water harvesting performance	Refs.
Wettability gradient (alternating hydrophilic/hydrophobic domains)	Namib Desert Beetle	Yarns weaving + dip‐coating	Bulked mesh (fiber diameter 10–20 µm)	1432.7 mg h^−1^ cm^−2^, ten cycles, fog flow rate: 300 L h^−1^	[47]
		Particle deposition on mesh + UV irradiation	Circular patterned fabric (pattern size 0.5‐2 mm)	609 mg h^−1^ cm^−2^, fog flow rate: 35 mL h^−1^	[48]
		Dipping + spray coating	Janus Fabric (fiber diameter 40 µm, surface nanorod diameter 240 nm)	2478.73 mg h^−1^ cm^−2^, ten cycles, fog flow rate: 300 mL h^−1^	[49]
		Spin coating + particle/micelle dispersion	Hierarchical buckled microchannels (1–10 µm)	1069 mg h^−1^ cm^−2^, humidifier air velocity of 1.2 m s^−1^ and volume of 0.072 m^3^ min^−1^	[50]
		Melting & micromolding	Micropillar (diameter 0.5–1.5mm, height 100–600 µm)	1251 mg h^−1^ cm^−2^, 20 cycles, fog flow from humidifier at RH 60 %	[51]
Laplace pressure gradient by curvature difference	Cactus Spine	Template method with photocuring process	Sharp needles on copper mesh (needle base diameter 1mm)	17.7 kg h^−1^ m^−2^, fog flow from humidifier (12 cm working distance)	[52]
		Dipping coating with thermal treatment	Nanocones (2–3 µm height, 200‐300 nm diameter) on 3D nylon fiber network	865 kg day^−1^ m^−2^, fog flow rate: 0.15m s^−1^	[53]
		3D Printing	Multibranched cluster spines (base: 200µm, height: 0.5–1 mm)	2 mg min^−1^ mm^−3^, 10 cycles, RH of 95 %	[55]
		3D Printing+ double inverse micromolding	3D hydrogel micro‐tree arrays (tip size: 20µm)	5000 mg h^−1^ cm^−2^, sustained over 20 months, fog flow rate: 1 m s^−1^	[57]
		Laser etching, + 3D cutting	Origami lattice (out frame size 60 mm x 60 mm)	0.6‐0.65g water in 15 mins, fog flow velocity (1.5–1.7 miles h^−1^)	[60]
Dual biomimetic design (wettability gradient + Laplace pressure gradient)	Spider silk	Parallel‐nozzle microfluidic technique	Single‐thread fiber and spindle‐knots (height:≈0.4mm, width: <0.2 mm)	4.47 g h^−1^ per single fiber, fog flow rate: 4mL min^−1^	[62]
		Dip‐coating nylon fibers + Rayleigh instability	Duo‐thread fibers and spindle‐knots (thread diameter: 20µm)	9.03 g h^−1^ cm^−2^·, fog flow rate: 120 mL h^−1^	[63]
		Dynamic interfacial spinning (DIS)	Multi‐thread fibers with periodic spindle‐knots (knot diameter: ≈45 µm, joint diameter: ≈7 µm)	0.4 µL per single knot in 24 s, fog flow rate: 0.17mL min^−1^	[64]
		Dip‐coating followed by mechanical stretching	Periodic spindle knots (width: 100–300 µm, height: 50–80 µm) with continuous axial microchannels (100–290 µm)	0.34 µL s^−1^ for a single fiber (3 cm in length), fog flow speed: 0.1 m s^−1^ RH of 95 %	[65]
Ternary biomimetic design (wettability + Laplace pressure + Gas‐liquid interfacial tension gradient)	Nepenthes Alata	Digital light 3D Printing	Multiscale curvature structure (ratchet teeth diameter 45 µm, arch peristome channel radius ≈2.3mm)	0.061 g^−1^ s^−1^ cm^−2^, RH of 95 %	[31]

## 3 Sorption‐Based Atmospheric Water Harvesting

### 3.1 Theoretical Basis

**Figure 7:**
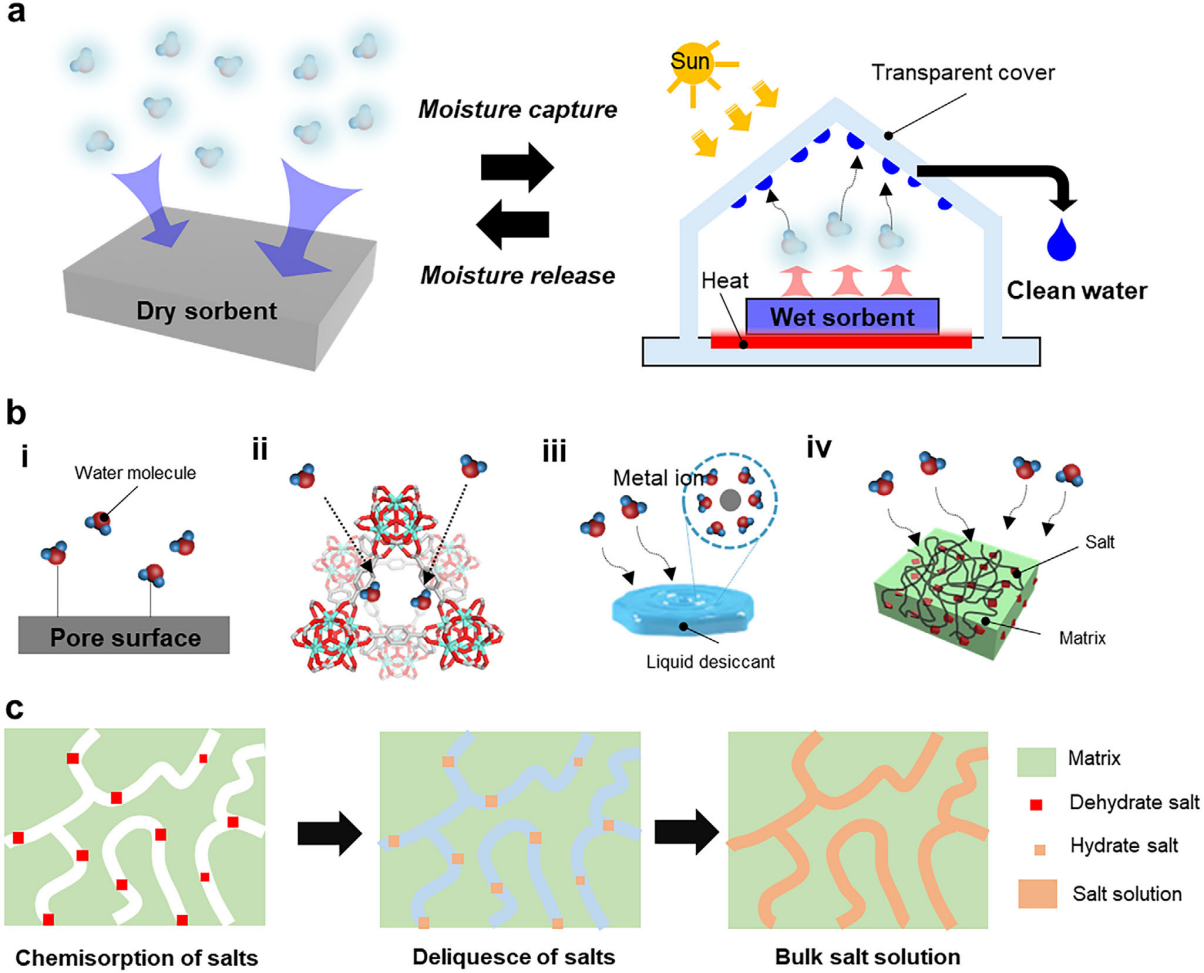
Working cycle and representative sorbent types for SAWH. a) Schematic of the AWH cycle, comprising moisture capture from air and release upon thermal or solar photothermal stimulation, followed by vapor condensation to yield liquid water. b) Schematics of different types of sorbents: i) physisorption on solid surfaces, ii) pore filling in crystalline porous frameworks of MOFs and COFs, iii) hygroscopic liquid desiccants via ion–dipole interactions, and iv) hydrogel–salt hybrid desiccant. c) Schematic of the three‐step sorption mechanism of the hygroscopic salt‐embedded composite.

SAWH harnesses the strong affinity of hygroscopic materials toward water vapor, enabling moisture capture under sub‐saturated humidity conditions, followed by release upon thermal stimulation.^[^ ^15^, ^19^ ^]^ The working cycle consists of two essential steps (**Figure** **7**a): 1) moisture capture, driven by physical adsorption, chemisorption, or absorption into hygroscopic media, and 2) moisture release, induced by external energy input, typically low‐grade heat (<100 °C) or solar photothermal heating followed by condensation to yield liquid water. The efficiency of this process is governed by key parameters of the sorbent, including equilibrium water uptake capacity (mass of water adsorbed per mass of dry sorbent, typically expressed in g g^−1^), sorption/desorption kinetics, regeneration energy demand, and cyclic stability under operational conditions.^[^ ^68^, ^69^ ^]^ These materials can be classified according to their sorption mechanisms and structural characteristics (Figure 7b), which are discussed in detail in the following section.

#### 3.1.1 Porous Solid Materials

Porous solid sorbents capture water vapor predominantly through physisorption driven by hydrogen bonding with surface functional groups or van der Waals interactions with pore walls (Figure 7b‐i).^[^ ^15^ ^]^ Their water uptake capacity is dictated by the distribution of pore sizes and the chemical nature of internal surfaces, which together define the shape of the sorption isotherm. Classical silica gels, composed of mesopores decorated with silanol groups, exhibit moderate uptake at intermediate RH but typically achieve below 0.3 g g^−1^ at 30 % RH.^[^ ^15^, ^70^ ^]^ Zeolites possess well‐defined micropores and strong electrostatic fields from framework cations, enabling significant adsorption even under arid conditions. However, the strong binding energy of water in zeolites necessitates high regeneration temperatures often above 150 °C, which restricts their integration into solar‐driven AWH systems.^[^ ^71^, ^72^ ^]^

Porous crystalline materials, such as MOFs and covalent organic frameworks (COFs), offer greater tunability in pore architecture and surface chemistry, allowing for low‐humidity water capture (Figure 7b‐ii).^[^ ^14^, ^73^ ^]^ Their sorption process can involve initial coordination of water molecules to open metal sites or polar linkers, multilayer adsorption along pore walls, and eventual capillary condensation within confined mesopores^[^ ^74^ ^]^. This precise control over the adsorption isotherm enables steep uptake transitions at RH as low as 10–30 %, which is advantageous for operation in desert climates. Representative examples include MOF‐801 (Zr‐fumarate framework), MIL‐101(Cr) (Cr‐terephthalate framework), and MOF‐303 (Al‐adeninate framework), which deliver equilibrium water uptakes of 0.25–0.8 g g^−1^ while regenerating under low‐grade heat (<90 °C).^[^ ^75^, ^76^, ^77^ ^]^ In COFs, hydrophobic–hydrophilic pore modulation provides a means to balance water uptake capacity and structural stability. The high crystallinity of these frameworks also facilitates rapid vapor transport when pore apertures are commensurate with the kinetic diameter of water molecules.^[^ ^73^ ^]^ Despite these advantages, hydrolytic instability, high cost of synthesis, and challenges in achieving kilogram‐scale production remain barriers to their widespread application.

#### 3.1.2 Liquid Desiccants

Liquid desiccants, typically concentrated solutions of hygroscopic salts such as lithium chloride (LiCl), calcium chloride (CaCl_2_), and lithium bromide (LiBr), capture water vapor through a three‐step process: 1) an initial chemisorption step, in which water molecules strongly coordinated with salt ions, 2) deliquescence, resulting in the formation of an aqueous salt solution, and 3) subsequent absorption of additional water vapor into the bulk solution (Figure 7b‐iii).^[^ ^78^, ^79^, ^80^, ^81^ ^]^ During absorption, progressive dilution reduces the salt concentration and lowers the vapor pressure depression, thereby gradually diminishing the driving force for uptake. As a result, these systems typically exhibit quasi‐linear isotherms rather than the step‐shaped profiles characteristic of solid sorbents.^[^ ^82^, ^83^ ^]^ Hygroscopic salts possess high water vapor sorption capacities (>0.5 g of water per gram of anhydrous salt) and operate over a wide range of RH, from low ≈10–20% RH to 100% RH.^[^ ^81^ ^]^ Regeneration is generally required at 60–120 °C, as strongly bound water molecules must overcome both hydrogen bonding and ion–dipole interactions, which depress the solution vapor pressure and thereby demand a higher thermal driving force for desorption. ^[^ ^78^, ^79^, ^81^, ^84^ ^]^ Among these salts, LiCl is particularly attractive due to its low deliquescence RH (≈10%) and moderate crystallization temperature (typically observed near 75–80 °C, depending on concentration), which allows for regeneration at relatively low temperatures (≈60–90 °C).^[^ ^79^, ^84^ ^]^ This makes LiCl highly compatible with solar photothermal nanomaterials for passive desorption strategies, whereas CaCl_2_ and LiBr generally require higher temperatures approaching 100–120 °C.^[^ ^78^, ^81^ ^]^ However, operation beyond the deliquescence point produces bulk salt solutions that hinder vapor diffusion, while recrystallization after desorption can lead to particle agglomeration and degraded absorption kinetics. Moreover, the liquid nature of concentrated salt solutions poses challenges in handling, corrosion, and salt carryover during regeneration. To address these limitations, recent efforts have focused on integrating hygroscopic salts into solid matrices such as hydrogels, porous scaffolds, or hybrid composites, which combine the high water affinity of salts with the structural stability and tailored transport pathways of solid frameworks.^[^ ^81^ ^]^

#### 3.1.3 Hygroscopic Salt‐Embedded Composite

Hygroscopic salt‐embedded composites, by embedding salts into porous hosts or a polymeric hydrogel matrix, are developed to overcome the inherent drawbacks of bulk liquid desiccants (Figure 7b‐iv). These composites exploit the high‐water affinity of salts while imparting mechanical stability and tailored transport channels from the matrix.^[^ ^81^, ^85^, ^86^, ^87^, ^88^ ^]^ A key design principle is how the salt is integrated into the host, as this determines performance in two fundamental ways: 1) the overall salt loading dictates the equilibrium water uptake capacity, 2) the uniform dispersion and size of salt govern the kinetics of absorption and desorption, as well as cycle stability. Because their vapor absorption behavior mainly originates from the embedded salts, these composites follow the same three‐step mechanism of chemisorption, deliquescence, and bulk absorption (Figure 7c).^[^ ^86^, ^89^ ^]^ From a thermodynamic perspective, confinement lowers the chemical potential of salts, thereby depressing the deliquescence RH and enabling capture of vapor at a low RH compared to using bulk salts.^[^ ^81^ ^]^ The kinetics of sorption are diffusion‐limited, such that smaller and more uniformly dispersed domains shorten transport pathways, while sorbent geometry (powders, films, fibers) further modulates mass transfer resistance and regeneration rates.^[^ ^81^, ^86^, ^90^ ^]^

The difference between porous media and hydrogel matrices in hygroscopic salt composites arises from how their structures regulate ion confinement and water diffusion. In porous media such as silica aerogel, porous carbon, or MOFs, the confined meso‐ or micropores (<50 nm) govern sorbate distribution and phase behavior through capillary and interfacial forces.^[^ ^91^ ^]^ When hygroscopic salts are introduced into such confined spaces, the limited pore volume and capillary tension restrict ion clustering and liquid accumulation within the porous network, a behavior observed experimentally in salt‐impregnated porous hosts such as SAPO‐34 (a crystalline silicoaluminophosphate zeolitic framework), and MIL‐101(Cr) where salt agglomeration and surface precipitation occur above ≈40–50 wt.% of salt loading of the total composite mass, indicating that pore confinement imposes a physical limit on salt loading and phase stability.^[^ ^92^, ^93^ ^]^ Capillary matrices, such as aerogels, porous active carbon felt, and cellulose nanofiber (CNF), enable much higher salt loading because the salt solution is retained by micrometre‐scale capillary forces rather than nanoconfinement. Their large pore volume and interconnected micrometre‐scale macroporous channels allow the liquid salt solution to adhere along the pore walls without requiring crystallization inside nanopores, achieving nearly complete salt loading (up to ≈97 wt.%). The overall sorption kinetics in these systems are governed by diffusive mass transfer, strongly dependent on the pore tortuosity (τ) and porosity (ε) of the matrix; lower τ and higher ε facilitate faster vapor diffusion within the porous network.^[^ ^94^ ^]^

Hybrid hydrogel desiccants, which integrate hygroscopic salt within a crosslinked hydrogel matrix, accommodate much higher salt contents (>80 wt.%) owing to their swelling ability. The polymer networks provide abundant functional groups that form ion–dipole and hydrogen‐bond interactions with both water and salt ions, allowing precise tuning of water uptake capacity (up to ≈8 g g^−1^ per gram of polymer), sorption/desorption kinetics, and even lowering of water release temperatures through thermoresponsive hydrogel network and self‐release liquid water without applying external heat.^[^ ^68^, ^81^, ^87^, ^95^, ^96^, ^97^ ^]^ They also allow molecular confinement of salts through the salting‐in effect of zwitterionic polymers, which accelerates sorption kinetics and improves retention.^[^ ^98^, ^99^ ^]^ Despite these advantages, hybrid desiccants remain relatively new, and their sorption mechanisms are still being studied. Recent studies have identified key parameters, including salt concentration, crosslink density, swelling ratio, film thickness, and diffusivity of the hydrogel network, that govern sorption kinetics in hybrid hydrogel desiccants.^[^ ^100^ ^]^ The sorption process involves coupled vapor and liquid transport within the hydrogel network, described by a two‐concentration model where the effective liquid diffusivity *D_l_* is expressed as

(8) $$D_{l}=\frac{2\left(1+\nu \right)G}{3\left(1-2v\right)\eta }k$$

where *k*, *G*, *η*, and *ν* denote the permeability, shear modulus, viscosity of water, and Poisson's ratio of the polymer network, respectively.^[^ ^100^ ^]^ These mechanistic insights lay the groundwork for translating material‐level control into structural designs for enhanced water harvesting.

Directly mimicking the complex hierarchical architectures found in natural hygroscopic systems at the material level is often impractical, owing to intrinsic limitations in composition, fabrication, and scalability. As a result, many recent advances have prioritized replicating structural motifs such as guided pathways, surface patterning, and hierarchical porosity that enhance vapor capture, transport, and release. This approach has proven particularly effective when applied to hybrid hydrogel desiccants, whose mechanical flexibility and processability allow the incorporation of intricate topologies with relative ease. In the following section, we highlight topology‐mimicking strategies that leverage such adaptable sorbents to guide sorption pathways and improve the overall efficiency of SAWH.

### 3.2 Topology‐Mimicking Structures for Guided Sorption Pathways

Highly efficient SAWH will not only offer high water capacity but also optimized mass transfer pathways that minimize vapor diffusion and liquid transport resistance.^[^ ^86^ ^]^ Natural systems, such as water transport in wood, transpiration in vascular plants, and the beaks of seabirds, serve as archetypes for hierarchical and anisotropic structures that efficiently guide water movement.^[^ ^101^, ^102^, ^103^ ^]^ By emulating these topological features, synthetic sorbent systems can achieve accelerated water uptake and release without compromising stability.

**Figure 8:**
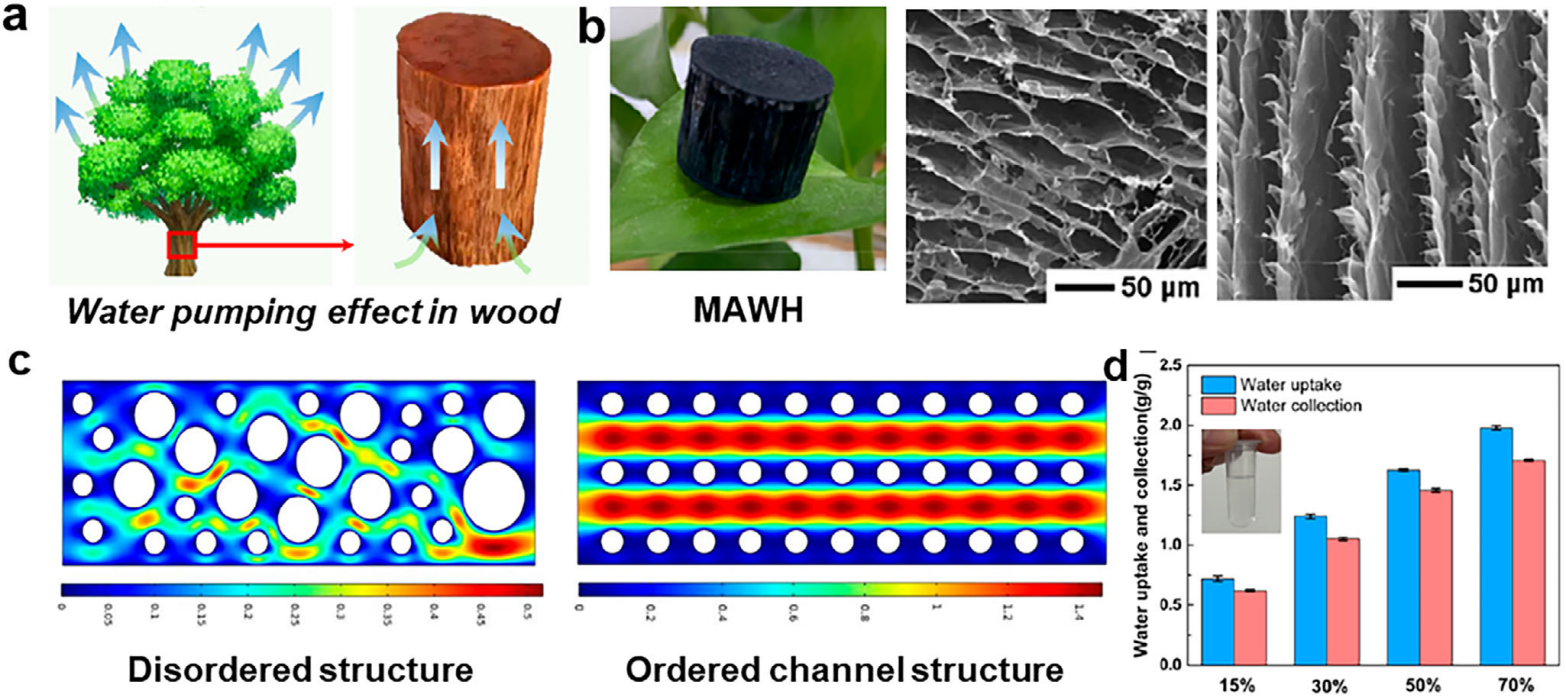
Ordered channel for directional vapor transport in SAWH. a) Schematic of the wood's natural water‐pumping effect. b) Photograph of the ultralight MAWH device and SEM images showing its side‐view morphology with ordered channel alignment. c) Simulated vapor transport behavior in (left) a disordered pore network and (right) an ordered channel array, with predicted transport rates. d) Measured water uptake and corresponding collection efficiencies of the MAWH at various RH, with solar‐driven desorption under one‐sun illumination. Reproduced with permission.^[^ ^105^ ^]^ Copyright 2024, American Chemical Society.

Pump effect in wood arises from its hierarchical vascular system, where vertically aligned xylem channels and porous cell walls form continuous capillary pathways for water uptake and transpiration. Driven by capillary pressure differences and reinforced by hydrogen bonding within the cellulose matrix, water spontaneously ascends and distributes throughout the tissue without external energy input.^[^ ^104^ ^]^ Inspired by this, Cai et al. report a dual absorption–adsorption networked MXene aerogel atmospheric water harvester (MAWH) that utilizes unidirectional ice‐templating to create vertically aligned microchannels with widths ranging from tens to hundreds of micrometers (**Figure** **8**a,b).^[^ ^105^ ^]^ The MXene–cellulose nanocrystal framework forms a dual hydrogen‐bonding absorption network, while confined LiCl provides a complementary adsorption network. This multiscale channel architecture reduces tortuosity and water nucleation barriers, enabling rapid vapor transport compared to disordered aerogels. COMSOL simulations predict vapor transport rates of up to 1.5 µm s^−1^ in the ordered channels, three times that of random structures (0.5 µm s^−1^), resulting in a more uniform vapor distribution across the porous matrix (Figure 8c). The device achieved stable cycling with water uptakes of 0.55–2.05 g g^−1^ at 15–70% RH and a collection efficiency of greater than 80% across all tested conditions (Figure 8d).

**Figure 9:**
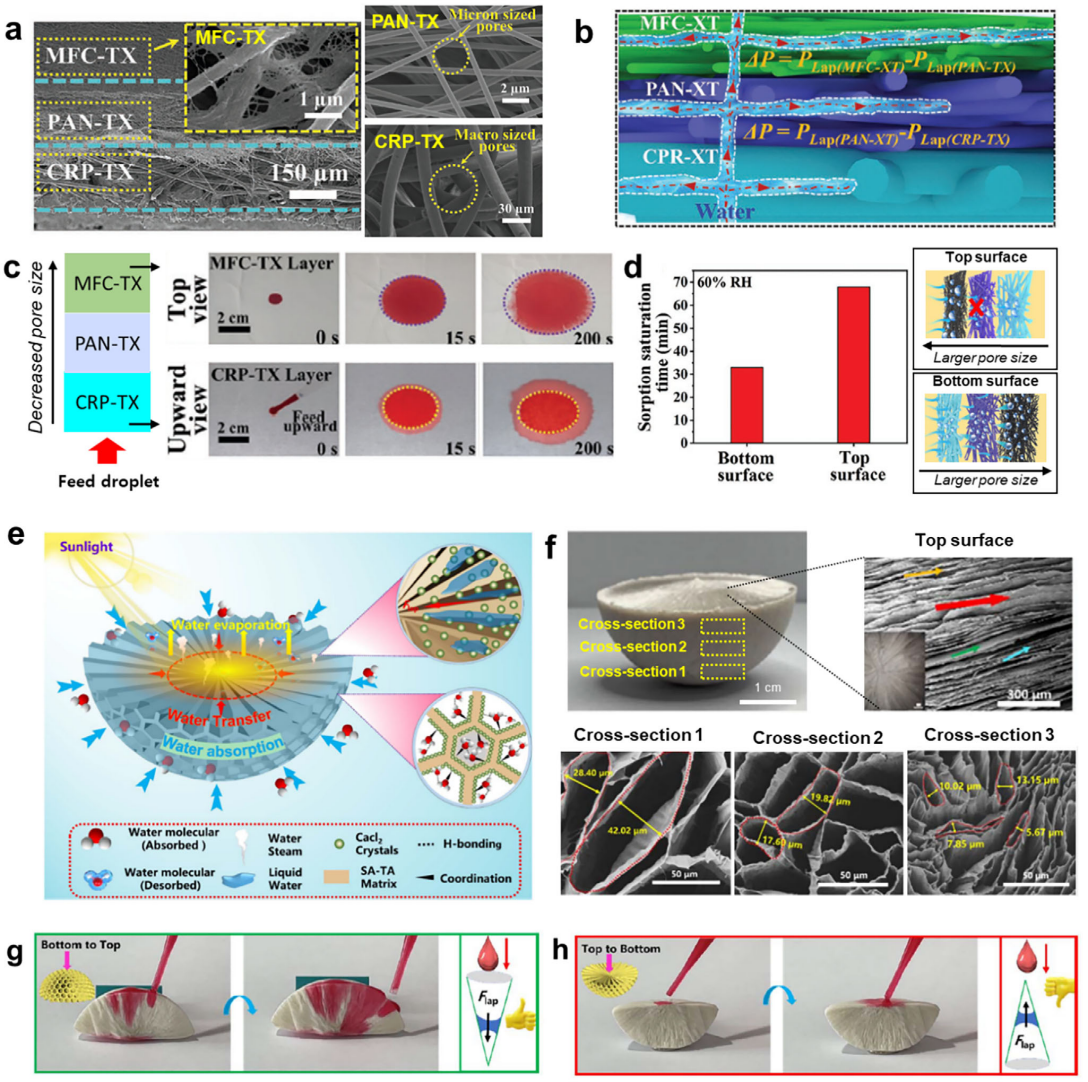
Nature‐inspired design for one‐way water transport. a) Hierarchically porous Murray membrane for vapor capture and liquid transport. Cross‐sectional SEM shows the multilayer structure, with a side view of the MFC‐TX layer; micron pores in CRP‐TX and macro pores in PAN‐TX. b) Schematic illustration of the directional water transport driven by Laplace pressure gradients across the pore‐size hierarchy. c) Dye penetration experiments visualizing anisotropic liquid spreading droplets fed from the CRP‐TX side, droplets diffusion upward showing MFC‐TX side spread more (purple‐dashed region) than those from the CRP‐TX side (yellow‐dashed region). d) Comparison of sorption saturation time at 60% RH depending on the vapor entering direction. Reproduced with permission.^[^ ^109^ ^]^ Copyright 2023, Wiley‐VCH. e) Schematic of hemispherical SA‐TA aerogel with centripetally aligned conical channels inspired by seabird beaks and insect eyes. f) The optical, top, and cross‐sectional SEM image of hemispherical aerogel. The hemisphere (top‐left) is fabricated using a copper mould (height = 3.5 cm, diameter = 5.5 cm, and 3.5 cm hemispheric depression). The scale bar is estimated based on the mould dimensions reported in the original publication. Optical images of a dyed water droplet transfer pathway: g) top to bottom, and h) bottom to top. Reproduced with permission.^[^ ^110^ ^]^ Copyright 2023, Royal Society of Chemistry.

Beyond single‐scale channel alignment, hierarchically porous membranes are designed to enable directional vapor and liquid transport across multiple length scales, thereby mitigating mass transfer limitations in SAWH. This design principle is inspired by Murray's law for vascular branching in biological systems, which states that the cube of the parent channel diameter equals the sum of the cubes of the daughter channels, thereby minimizing hydraulic resistance and energy loss across hierarchical networks.^[^ ^106^, ^107^ ^]^ This law has been further generalized in a hierarchically porous system,^[^ ^108^ ^]^ expressed as

(9) $${r_{0}}^{a}=\frac{1}{1-X}\sum_{i}{r_{i}}^{a}$$

where *r*_0_ and *r_i_* denote the radii of the parent and daughter channels, respectively, *X* accounts for mass loss or gain during the transport, and *a* = 3 for laminar flow, *a* = 2 for diffusion/ion transfer. This relation provides a scaling rule indicating that hierarchical pore architectures facilitate faster vapor diffusion and liquid flow by minimizing transport resistance across multiple length scales. In line with this framework, Xia et al. fabricate a tri‐layer hygroscopic fibrous membrane composed of a macro‐porous clean‐room paper layer (CRP‐TX, average pore size ≈45 µm), a micro‐porous polyacrylonitrile layer (PAN‐TX, ≈1.6 µm), and a sub‐micron fibrillated cellulose layer (MFC‐TX, ≈450 nm) (**Figure** **9**a).^[^ ^109^ ^]^ This ordered gradient in pore size generates Laplace pressure differentials, governed by the Young–Laplace relation (Equation 6), which drive water from larger to smaller pores, reducing mass‐transfer resistance across the membrane (Figure 9b). They validate this mechanism by dye penetration experiments, which directly visualize directional liquid transport (Figure 9c). When a red‐dyed droplet (100 µL) is fed from the MFC‐TX side with smaller pores, the liquid rapidly penetrates upward and spreads widely (purple‐dashed region), whereas feeding from the CRP‐TX side with larger pores results in slower diffusion and a more confined wetted area (yellow‐dashed region) (Figure 9c). Consequently, this tri‐layer membrane reduces sorption saturation time by more than half compared to reversed orientation (Figure 9d). This directional pumping behavior demonstrates that pore‐size hierarchy, rather than gravity, governs water movement. As a result, the PPy–COF@Trilayer–LiCl composite achieves rapid water uptake of 0.77–2.56 g g^−1^ across 30–80% RH within 50 min, and releases >95 % of absorbed water within 10 min under one sun irradiation.

As an alternative to hierarchical branching dictated by Murray's law, Bu et al. introduce another topological strategy by translating the cone‐shaped beak geometry of seabirds into a hemispherical sodium alginate–tannic acid (SA–TA) hydrogel with radially aligned microscale conical channels, fabricated through multidirectional ice‐templating (Figure 9e).^[^ ^110^ ^]^ This design not only reproduces the one‐way pumping effect of asymmetric capillary ratchets but, by further adopting the centripetal arrangement motif of insect compound eyes, enables radial ordering that guides absorbed vapor inward for directional liquid transport and solar‐driven release (Figure 9e). The resulting aerogel features a gradient in microchannel size, decreasing from the outer edge toward the center (Figure 9f), thereby establishing Laplace pressure differentials (see Equation 6) that facilitate unidirectional water motion along the narrowing pathways. As a result, when the dyed droplets are introduced from the bottom side, they rapidly migrate upward along the channels (Figure 9g), whereas feeding from the top leads only to limited spreading without inward pumping (Figure 9h). Due to the one‐way water pumping effect, this bioinspired sorbent achieves water uptake up to 2.29 g g^−1^ at 90 % RH and accelerates saturation within 2 h, far exceeding that of raw CaCl_2_ powder or alginate alone. The photothermal coating enables rapid release at ≈1.77 kg m^−2^ h^−1^ under one sun, while outdoor tests demonstrate daily yields of ≈3.7 L m^−2^, sufficient for potable use.

### 3.3 Hierarchical Confinement Strategy for Accelerated Sorption Kinetics

**Figure 10:**
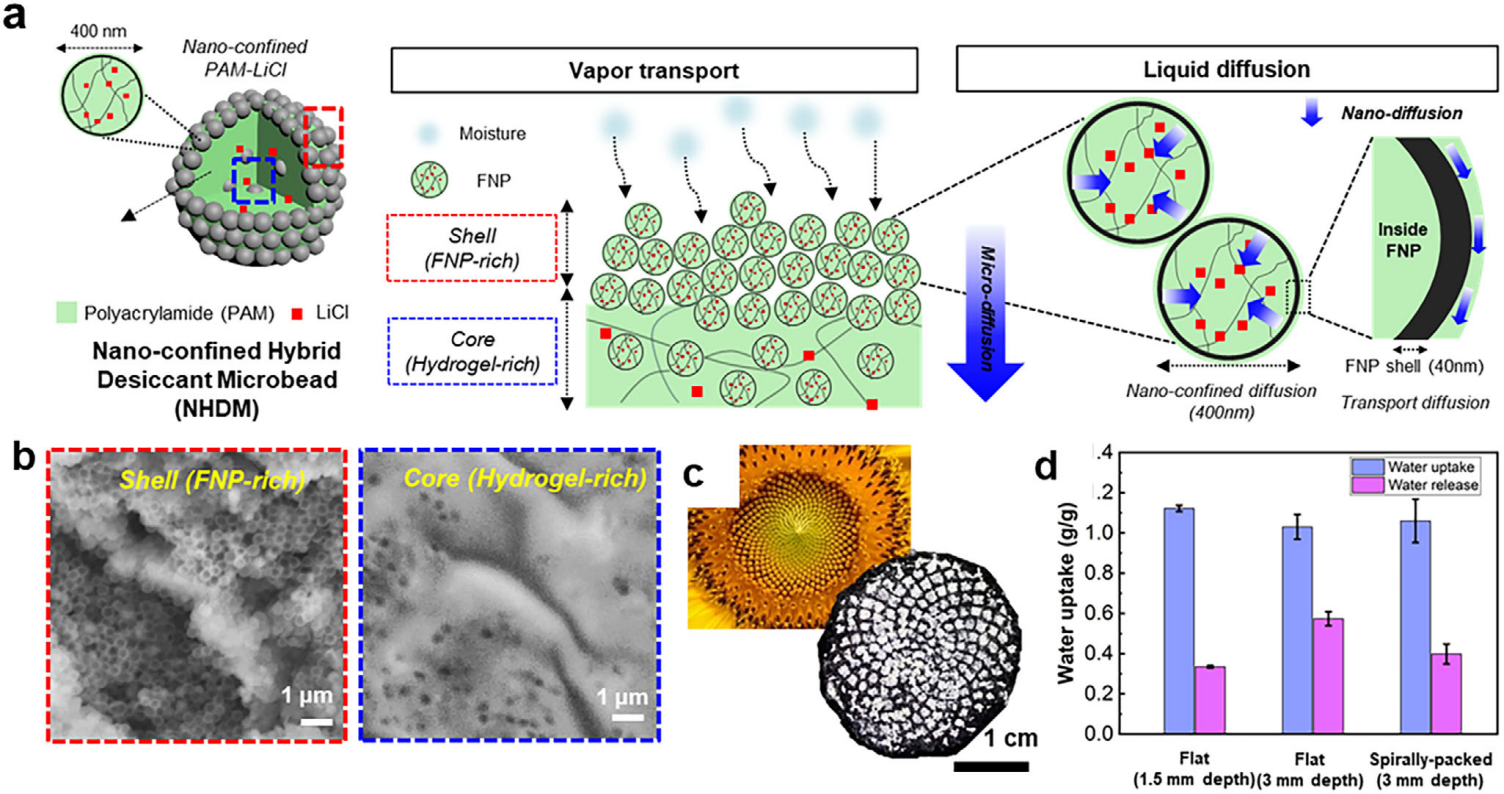
Hierarchically confined hybrid desiccant microbeads and bio‐inspired spiral packing for enhanced SAWH efficiency. a) Schematic illustration of raspberry‐like nano‐confined hybrid desiccant microbead (NHDM) showing the nanoparticle‐rich shell and hydrogel‐rich core structure that facilitates moisture diffusion. b) Representative SEM images highlighting the surface morphology (shell region) and interior (core region) of NHDMs. c) Photograph of a sunflower with seeds arranged in a Fibonacci spiral, alongside a device disk featuring spirally packed NHDMs that mimic this natural geometry. d) Comparison of sorption‐desorption performance between flat and spiral‐packed NHDMs, measured at 65% RH and 21 °C for uptake and under 1‐sun irradiation for release. Reproduced with permission under the terms of the Creative Commons CC BY‐NC license.^[^ ^111^ ^]^ Copyright 2025, The Authors. Published by Wiley‐VCH.

While topology‐mimicking structures optimize transport pathways through ordered and anisotropic channels, their structural complexity makes large‐scale implementation challenging. Lee et al. report a hierarchical confinement strategy for a hybrid desiccant that takes a different approach by engineering sorbents into multiscale particulate architectures to shorten diffusion lengths and enlarge vapor‐sorbent interfaces.^[^ ^111^ ^]^ They develop a highly scalable raspberry‐like microbead sorbent via the Pickering emulsion assembly of polyacrylamide–LiCl (PAM–LiCl) hybrid hydrogels, featuring a nanoparticle‐rich shell and a hydrogel‐rich core (**Figure** **10**a,b). This dual‐scale confinement, with LiCl localized within nanoscale hydrogel networks and microscale bead morphology, not only shortens diffusion pathways but also markedly increases the effective surface area exposed to vapor, thereby accelerating sorption kinetics compared to bulk hydrogel‐salt composites (Figure 10a). The microbeads achieved ≈1.11 g g^−1^ uptake within 60 min at 65% RH, reaching 80% saturation in only 71 min, 4.4 times faster than the bulk counterpart. Besides material‐level design, a bio‐inspired packing architecture was introduced by arranging the microbeads on spirally engraved disks coated with PPy, which mimics the Fibonacci sequence of sunflower seeds (Figure 10c). This geometric strategy simultaneously increased the bead loading density per unit area and minimized self‐shading of the photothermal PPy coating. Compared to flat‐packed configurations, the spiral arrangement improved the AWH efficiency by ≈34% and enabled higher daily water yields under both laboratory and outdoor conditions (Figure 10d).

### 3.4 Core–Shell and Bilayer Structures for Salt Leakage Prevention

Salt leaching is a primary durability concern in hygroscopic salt‐based sorbents, as the dissolution and migration of salt under humid conditions can gradually diminish water uptake capacity and lead to environmental contamination. To mitigate this issue, core–shell and bilayer architectures have been designed to physically confine the salt within a stable matrix while maintaining high vapor permeability.

**Figure 11:**
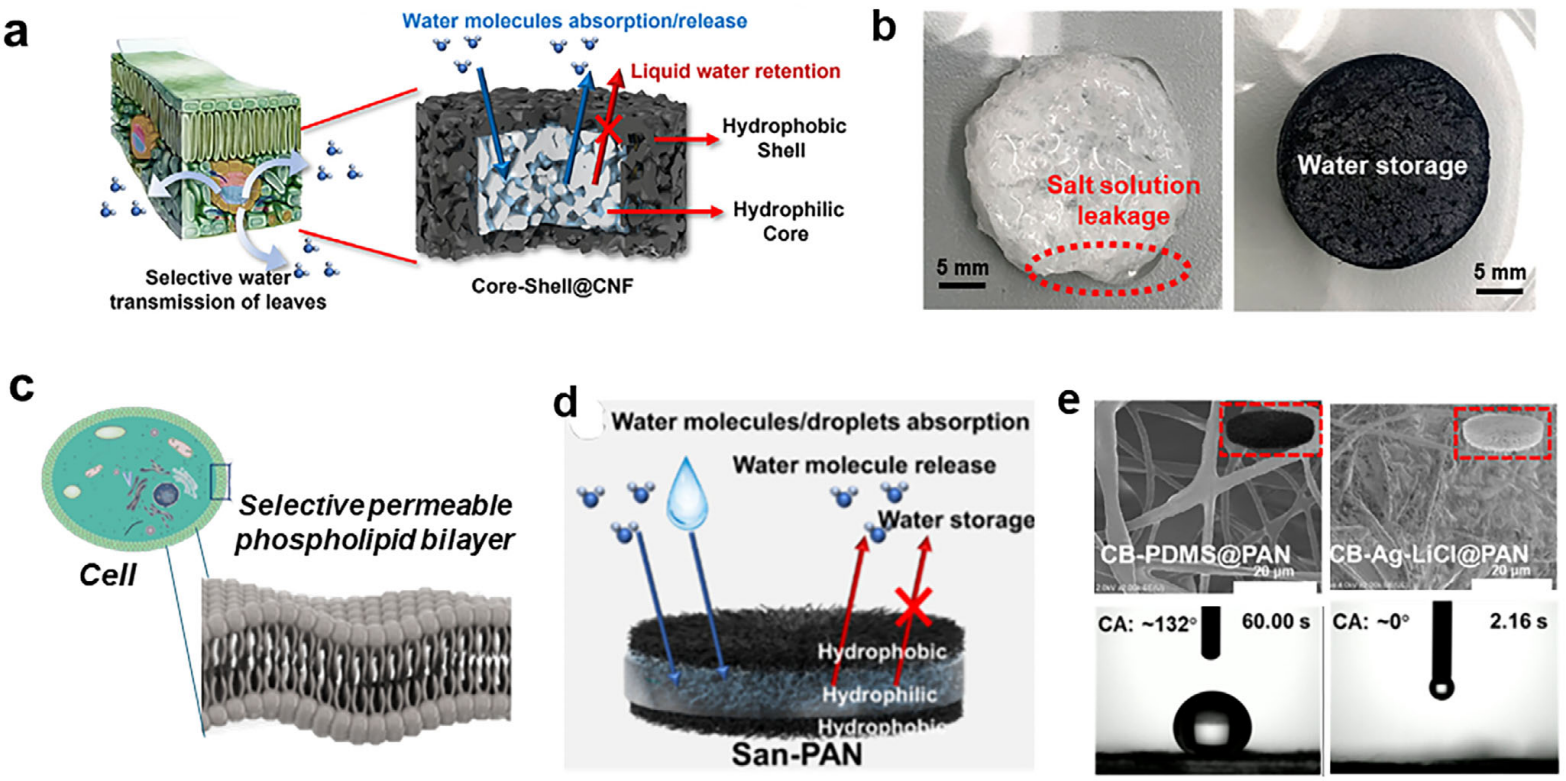
Bilayer structures for preventing salt leakage. a) Leaf‐inspired selective water transmission and schematic of the Core–Shell@CNF architecture. b) Photographs comparing water uptake and leakage behaviors of LiCl@CNF and Core–Shell@CNF at 25 °C and 90 % RH. Reproduced with permission.^[^ ^114^ ^]^ Copyright 2023, Elsevier. c) Illustration of selective permeability in a phospholipid bilayer. d) Water capture and release mechanism of the San‐PAN structure. e) SEM images show the pore size of PAN, CB@PAN, CB–PDMS@PAN, and CB‐Ag‐LiCl@PAN, with corresponding water contact angles for CB‐PDMS@PAN and CB‐Ag‐LiCl@PAN. Reproduced with permission.^[^ ^116^ ^]^ Copyright 2024, American Chemical Society.

Inspiration comes from the core–shell structures naturally found in plant leaves, where the waxy cuticle layer protects inner mesophyll tissues against dehydration and oxidation, while stomata selectively mediate gas exchange.^[^ ^112^, ^113^ ^]^ Inspired by this dual functionality, Yu et al. report a bio‐inspired core–shell structured CNF aerogel that mimics the selective water transport in plant leaves (**Figure** **11**a).^[^ ^114^ ^]^ The hydrophilic CNF core is impregnated with LiCl for strong moisture uptake, while the outer CNF shell is modified with PDMS and carbon black (CB), imparting hydrophobicity (water contact angle ≈120°) and photothermal activity. A minimum shell thickness of ≈1 mm is required to avoid structural defects that cause leakage. The pore size of the shell is tuned to ≈0.75–1 µm, which lies well above the mean free path of water molecules (≈57 nm) to ensure vapor transmission, yet far below the critical pore size (≈1.49 mm) at which liquid saline can escape under hydrostatic pressure. This design, therefore, selectively permits gaseous water transport while physically confining the salt solution within the core. The result is stable water absorption and release without salt loss, as evidenced by consistent performance over multiple sorption–desorption cycles compared with severe leakage in conventional salt‐loaded aerogels (Figure 11b). Benefiting from vertically aligned CNF pores that accelerate vapor transport, Core–Shell@CNF achieves ≈1.16 g g^−1^ uptake within 2 h at 25 °C and 90% RH.

Although the core–shell CNF aerogel successfully confines salt within a protective barrier, the single‐layer shell limits structural robustness under prolonged cycling and does not fully integrate efficient thermal management for rapid water release. To overcome these limitations, Yu et al. report a bilayer‐inspired sandwich‐structured PAN nanofibrous membrane (San‐PAN), drawing inspiration from the phospholipid bilayer in cell membranes, where hydrophilic heads and hydrophobic tails assemble into a sandwich‐like architecture that controls the transport of nutrients and water (Figure 11c).^[^ ^115^, ^116^ ^]^ The San‐PAN membrane consists of a hydrophilic PAN middle layer loaded with LiCl and coated with Ag nanoparticles to enhance thermal conductivity, sandwiched between two hydrophobic PAN outer layers modified with carbon black (CB) and PDMS (Figure 11d). Electrospinning constructs the fibrous PAN scaffolds, providing a highly porous and mechanically stable network for vapor transport. Subsequent Ag‐LiCl impregnation uniformly distributes hygroscopic salt while simultaneously improving thermal conductivity through Ag nanoparticles, thereby facilitating both high water uptake and rapid desorption. Finally, CB‐PDMS dip‐coating endows the outer layers with strong hydrophobicity and photothermal activity, enabling selective vapor transmission, effective solar heating, and reliable salt confinement. SEM and contact angle measurements confirm distinct fibrous morphologies and a sharp wettability contrast (≈132° for CB‐PDMS@PAN, whereas ≈0° for CB‐Ag‐LiCl@PAN) (Figure 11e). The hydrophobic layers feature micron‐sized pores (≈9–11 µm) with ≈200 µm thickness, large enough to permit water vapor (≈0.4 nm) diffusion yet small and robust enough to block liquid brine, thereby confining the salt solution through a combination of size exclusion, capillary resistance, and dual‐layer integrity (Figure 11e). As a result, the bilayer structure maintains stable operation without salt leakage even after 50 cycles, achieving rapid sorption–desorption cycling and water uptakes ranging from 1.66 to 4.08 g g^−1^ over 30–90 % RH.

### 3.5 Liquid Water Self‐Releasing Systems

**Figure 12:**
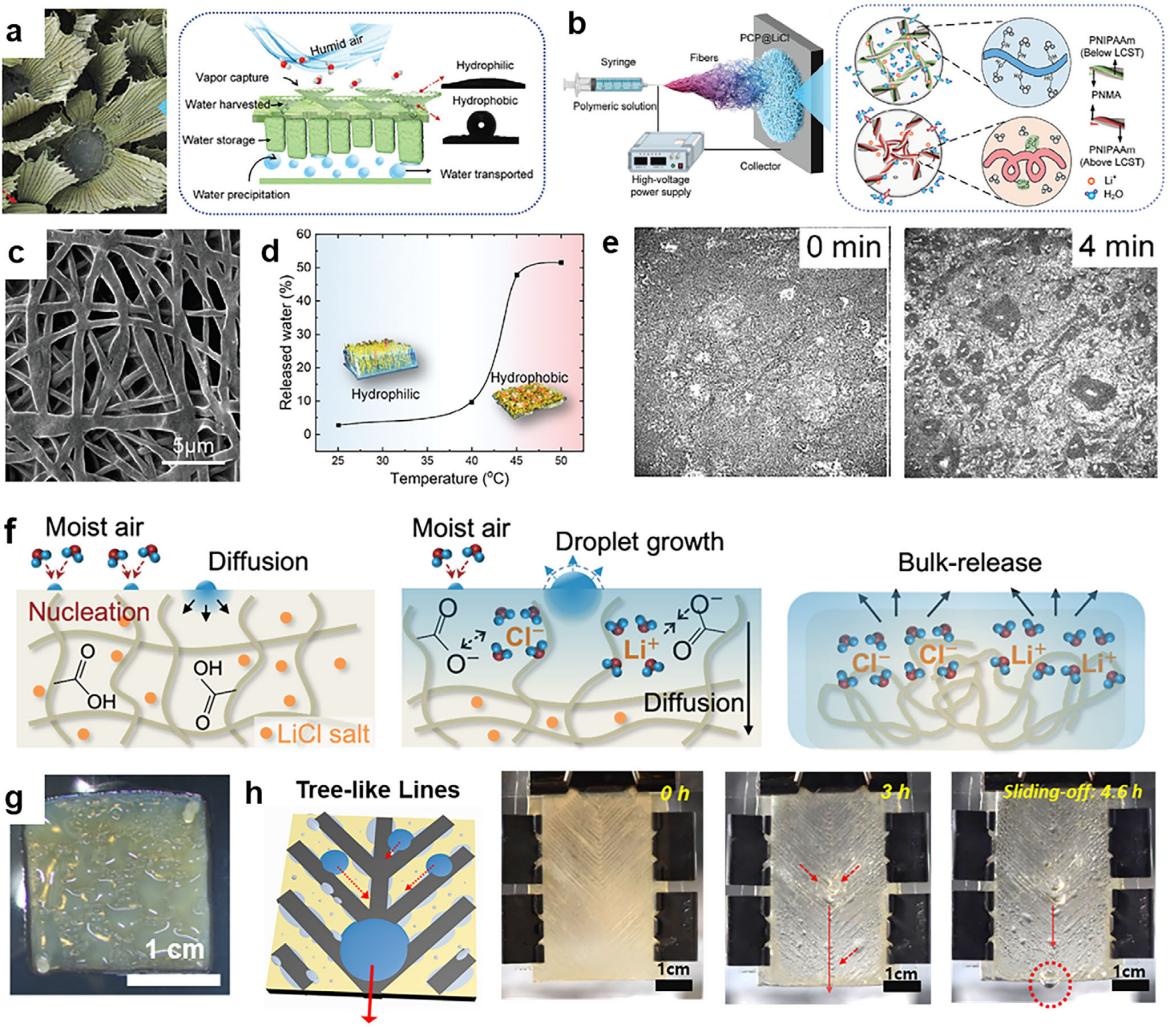
Releasing liquid water from hybrid hydrogel desiccant films. a) Bio‐inspired moisture‐responsive behavior of *Tillandsia* leaves and water transport in the leaf blade. b) Electrospinning fabrication of thermo‐responsive PCP@LiCl fibers and schematic of hydrophilic–hydrophobic transition. c) SEM and cryo‐EM images of PCP@LiCl during adsorption. d) Water contact angle changes with temperature. e) Time‐lapse images of water release from PCP@LiCl. Reproduced with permission.^[^ ^120^ ^]^ Copyright 2024, Wiley‐VCH. f) Schematic of moisture uptake and self‐release in PAA–LiCl films. g) Water droplets formed on a PAA–LiCl hydrogel. h) Guided droplet movement and detachment on PAA–LiCl films with PS tree‐like patterns. Reproduced with permission.^[^ ^97^ ^]^ Copyright 2023, Wiley‐VCH.

Conventional SAWH systems produce liquid water indirectly, as discussed in Section 3.1: sorbents first capture vapor, which is subsequently released and condensed on a cooled surface. This approach inherently requires heat input for regeneration and suffers from condensation losses, since not all released vapor can be fully collected. To overcome these limitations, an emerging strategy aims to bypass the vapor‐condensation step altogether by engineering sorbents that can directly expel liquid water. Representative efforts include utilizing thermo‐responsive polymer matrices, which undergo a hydrophilic‐to‐hydrophobic transition above their lower critical solution temperature (LCST, the threshold at which polymer–water interactions switch from soluble to insoluble).^[^ ^117^ ^]^ Notable examples are poly(N‐isopropylacrylamide) (PNIPAAm, LCST ≈32 °C) and hydroxypropyl cellulose (HPC, LCST ≈40–45 °C). Water captured and stored in them can be released under mild conditions (≈40 °C).^[^ ^90^, ^118^, ^119^ ^]^ Wang et al. further combined these thermo‐responsive polymers with the leaf‐inspired architecture of *Tillandsia* to develop a bioinspired liquid‐releasing system.^[^ ^120^ ^]^ *Tillandsia*, commonly known as air plants, survive without soil by harvesting moisture directly from the atmosphere.^[^ ^121^ ^]^ Their leaves are covered with specialized cells (trichomes) that rapidly absorb and channel water into storage tissues. The overall leaf architecture combines a hydrophilic adaxial surface with a hydrophobic abaxial surface, enabling efficient droplet capture and directional transport toward the plant's interior.^[^ ^122^, ^123^ ^]^ Inspired by *Tillandsia* plants (**Figure** **12**a), Wang et al. report a nanofibrous hygroscopic membrane (PCP) comprising interpenetrating networks of PNIPAAm and poly(N‐methylacrylamide) (PNMA), which reinforced the mechanical stability of the hydrogel matrix, while embedded carbon nanotubes enhanced solar–thermal conversion.^[^ ^120^ ^]^ The fibers were impregnated with LiCl to obtain PCP@LiCl, enabling efficient water uptake under low humidity and temperature‐triggered liquid water release. The membrane was fabricated via coaxial electrospinning (Figure 12b), forming an interconnected porous network (Figure 12c). Below LCST, PNIPAm remains hydrophilic, retaining liquid water. When above the LCST, it becomes hydrophobic, facilitating rapid water expulsion (Figure 12d). This design enables visible water release within 4 min of solar heating (Figure 12e). It exhibits water uptake capacities of 0.43, 0.89, and 1.48 g g^−1^ at 15 %, 30 %, and 60 % RH, respectively, reaching saturation within 2 h at 25 °C under low RH (15–30 %), and releasing ≈50 % of the absorbed water directly as liquid within 5 min under solar irradiation.

While thermo‐responsive hydrogels still require heating to reach the LCST for water release, Nah et al. demonstrate an alternative approach by combining an anionic polymer network, poly(acrylic acid) (PAA), with LiCl to create a film capable of simultaneously capturing vapor and producing liquid water directly under ambient conditions (Figure 12f).^[^ ^97^ ^]^ They reveal that the liquid‐water release originates from interactions between the anionic charged PAA chains and Li⁺/Cl^−^ ions, which promote water accumulation and subsequent expulsion. The water‐releasing behavior proceeds via two distinct stages: 1) surface release, where water droplets nucleate and grow on the hydrogel surface due to differences in nucleation and diffusion rates, and 2) bulk release, triggered by polymer chain collapse that expels water stored within the hydrogel network (Figure 12f,g). To further enhance droplet growth and guide directional transport, hydrophobic tree‐like patterns are introduced on the hydrogel surface, exploiting edge effects and facilitating the acceleration of water movement (Figure 12h). This pattern increases the water removal rate from the film nearly three times that of the pristine film.

These strategies, including topology‐guided channels, hierarchical confinement, core–shell architectures, and liquid‐water‐releasing systems, represent diverse bioinspired approaches for SAWH. Representative examples, along with their inspirations, fabrication methods, structural features, and performances, are summarized in **Table** **2**.

**Table 2:** Summary of recent SAWH systems with bioinspired design strategies.

Objective	Inspiration	Method	Materials	Structure/Scale	Performance	Refs.
Topology‐guided for improving transport efficiency	Wood xylem	Ice‐templating	MXene‐cellulose / LiCl	Vertically aligned μ‐channels (10–100 µm)	0.555–2.05 g g^−1^ at 15–70% RH within 10 h	[105]
	Murray's law	Tri‐layer fibrous membrane	CRP‐PAN‐MFC / LiCl	Trilevel membrane with pore gradient: 45 µm, 1.6 µm, and 450 nm	0.77–2.56 g g^−1^ at 30–80% RH within 50 min	[109]
	Seabird beak (cone) & Insect eyes (hemisphere)	Freeze‐casting	SA‐TA / CaCl_2_	Hemispherical, radial conical μ‐channels (≈5‐40 µm)	2.29 g g^−1^ at 90 % within 2h	[110]
Faster kinetics & Increase loading density	Raspberry & Sunflower spiral packing	Pickering emulsion	PAM / LiCl	Microbead (≈620 µm) nanoconfined PAM‐LiCl (≈400nm)	0.57–1.11 g g^−1^ at 30–65% RH within 60 min	[111]
Salt leakage prevention	Core‐shell structures in plant leaves	Freeze‐drying	Core: CNF / LiCl Shell: CB‐PDMS	Core (hydrophilic, ≈10mm) Shell (Hydrophobic, ≈1mm)	1.16 g g^−1^ at 90 % within 2h Stable 10 cycles	[114]
	Phospholipid bilayer	Electrospinning Dip‐coating	Inner: CB‐Ag‐LiCl@PAN Outer: CB‐PDMS@PAN	Outer hydrophobic layer (≈9‐11µm with ≈200µm thickness)	1.66 to 4.08 g g^−1^ over 30‐90 % RH within 100 min Stable 50 cycles	[116]
Facilitating liquid water release	Tillandsia leaves	Electrospinning	PNIPAm‐PNMA / LiCl	thermo‐responsive switching mimics Tillandsia adaxial (hydrophilic) / abaxial (hydrophobic) sides 500nm fibers, porous network with 1‐5µm pores	0.43 to 1.48 g g^−1^ over 15‐60 % RH within 2h ≈50% of absorbed water released directly as liquid within 5 min under 1 sun irradiation	[120]
Guided liquid release	Tree branches	Spray coating of PS to form a hydrophobic tree‐like pattern	PAA / LiCl, hydrophobic pattern of PS	Microchannel ≈500µm	Self‐release of liquid water without heat	[97]

CRP: clean‐room paper, PAN: polyacrylonitrile, MFC: micro‐fibrillated cellulose; SA‐TA: sodium alginate–tannic acid; PAM: polyacrylamide; CNF: cellulose nanofiber; CB: carbon black; PDMS: polydimethylsiloxane; PNIPAm: poly(N‐isopropylacrylamide); PNMA: poly(N‐methylacrylamide); PAA: polyacrylic acid; PS: polystyrene.

## 4 Conclusion and Perspectives

This review highlights the design principles and material strategies underpinning nature‐inspired AWH systems, with a particular focus on how structural motifs and hierarchical materials engineering accelerate atmospheric water capture, droplet transport, and energy‐efficient release. Fog‐collection systems inspired by beetle backs, cactus spines, spider silk, and Nepenthes peristomes demonstrate how wettability gradients, Laplace pressure differentials, and anisotropic channel architectures can enhance droplet nucleation and directional transport. SAWH systems, in turn, leverage porous solids, crystalline frameworks, liquid desiccants, and hybrid hydrogel‐salt composites, often integrated with bio‐inspired topologies, to overcome the intrinsic trade‐offs between uptake capacity, sorption kinetics, and regeneration energy. Collectively, these advances demonstrate the power of biomimetic principles in bridging fog and vapor‐based AWH mechanisms.

Among these, recently developed sorbents for utilizing atmospheric vapor represent a significant breakthrough, offering exceptionally high water uptake across a wide range of humidity compared to conventional solid adsorbents.^[^ ^15^, ^81^ ^]^ Notably, because vapor sorption is not constrained by the humidity resistance at different geometric locations, these materials enable continuous water production even in arid areas.^[^ ^90^, ^124^, ^125^ ^]^ However, despite their impressive performance on a laboratory scale, practical translation is still hindered by limited scalability and incomplete mechanistic understanding. To accelerate progress, the field must move beyond material‐centric discovery toward device‐level design and operational strategies that maximize productivity in realistic environments. Recently, a MOF‐based water harvester^[^ ^125^ ^]^ and a solar‐driven drum‐type harvester that integrates bio‐based CAL gels^[^ ^89^ ^]^ have demonstrated that increasing the cycling frequency in a tunable, portable, and scalable platform can significantly improve water yield under outdoor conditions. In parallel, all‐day operation has been achieved through multicyclic sorption‐desorption with hybrid desorption (solar plus low‐grade heat), thereby increasing daily throughput.^[^ ^126^ ^]^

A recent study further exemplifies this bridging concept, where a spider‐silk‐inspired MOF–hydrogel fiber integrates MOFs within a zwitterionic hydrogel matrix to stabilize hygroscopic salts and enhance low‐humidity vapor uptake, while adopting spindle‐knot morphologies characteristic of natural spider silk.^[^ ^127^ ^]^ Although originally developed for sorption‐based water harvesting, its spider‐silk‐like geometry, previously utilized for fog collection, demonstrates that such bioinspired structural designs can also be effectively applied to SAWH systems.

Nonetheless, large‐scale deployment of both fog‐ and sorption‐based AWH materials remains constrained by the complexity and cost of manufacturing. Many high‐efficiency designs depend on sophisticated hierarchical architectures, micro/nano‐structured fog collectors, or polymeric sorbents fabricated through processes such as lithography, electrospinning, 3D printing, or freeze‐drying, which are difficult to reproduce uniformly or cost‐effectively at scale. To bridge this gap, future research should pursue scalable fabrication strategies that retain structural fidelity while minimizing energy input and cost. Promising directions include roll‐to‐roll imprinting, templated casting, and self‐assembly for fog‐harvesting surfaces, as well as powderized microgels and bead‐based sorbents that can offer controlled porous morphology to achieve high vapor‐sorption kinetics while being more compatible with large‐batch or continuous production.^[^ ^90^, ^111^ ^]^ Such efforts are essential to transform laboratory prototypes into deployable and affordable AWH systems.

Future research in AWH should thus transcend the conventional separation between fog collection and vapor sorption, enabling synergistic and continuous operation across diverse environmental conditions. One potential direction is the integration of fog‐harvesting and sorption‐based vapor‐harvesting materials into a coupled system. In such a hybrid configuration, hygroscopic sorbents capture and release vapor during low‐humidity or nighttime operation, which is subsequently condensed and collected by adjacent fog‐harvesting layers featuring optimized surface‐energy gradients and capillary‐driven transport. This sequential process could allow for efficient utilization of both adsorptive and condensational mechanisms, minimizing evaporative losses and maintaining continuous water generation throughout diurnal humidity fluctuations.

An even more ambitious direction could lie in the integration of both water vapor and liquid‐droplet harvesting functions within a single, multifunctional material for continuous atmospheric water harvesting under “all‐day, all‐weather” conditions. This approach dynamically adapts to environmental humidity and light conditions, functioning as vapor absorbers at night and fog collectors during the day. Janus or gradient‐structured materials that couple a hydrophilic sorptive matrix with a textured surface could simultaneously promote vapor uptake, condensation, and directional droplet transport. Stimuli‐responsive polymers or MOF–hydrogel hybrids capable of modulating porosity, polarity, or surface energy under environmental stimuli, such as sunlight, temperature, or humidity, may enable self‐regulated switching between sorption and condensation modes. Integration with photothermal or radiative cooling components could further enable diurnal cycling, sustaining water production across humid and arid conditions. The challenges are how to maintain mechanical and chemical stabilities under repeated hydration–dehydration cycles, minimize salt leakage, and balance sorption kinetics with wetting dynamics issues, which demand multiscale material designs, advanced heat‐mass transfer modeling, and systematic field validation under natural conditions.

Sustainability considerations are also critical. The use of biopolymer scaffolds, such as cellulose, alginate, and lignin, provides renewable and biodegradable carriers that confine salts, mitigate leakage, and reduce environmental burden.^[^ ^89^, ^128^ ^]^ These bio‐derived matrices exemplify how high performance and ecological compatibility can advance in parallel, which will be essential for large‐scale adoption.

Considering these insights, AWH emerges not only as a scientific frontier but also as a transformative pathway for global water sustainability. These systems uniquely harness a ubiquitous and renewable reservoir from atmospheric water. Beyond reviewing advances in materials, structures, and device integration, this article emphasizes how biological inspiration provides guiding principles for engineering solutions. Looking ahead, the continued convergence of sorbent chemistry, scalable fabrication, and system‐level optimization will be crucial to realizing the full societal impact of AWH. With these efforts, next‐generation AWH platforms are poised to evolve into reliable, sustainable technologies that extend beyond water supply to multifunctional roles in energy and environmental management.

## Conflict of Interest

The authors declare no conflict of interest.

## Author Contributions

Y.L. and S.F. contributed equally to this work. The order of authors does not reflect the extent of their contribution.
